# Compassion: From Its Evolution to a Psychotherapy

**DOI:** 10.3389/fpsyg.2020.586161

**Published:** 2020-12-09

**Authors:** Paul Gilbert

**Affiliations:** Centre for Compassion Research and Training, College of Health and Social Care Research Centre, University of Derby, Derby, United Kingdom

**Keywords:** compassion, psychotherapy, evolution, caring, biopsychosocial

## Abstract

The concept, benefits and recommendations for the cultivation of compassion have been recognized in the contemplative traditions for thousands of years. In the last 30 years or so, the study of compassion has revealed it to have major physiological and psychological effects influencing well-being, addressing mental health difficulties, and promoting prosocial behavior. This paper outlines an evolution informed biopsychosocial, multicomponent model to caring behavior and its derivative “compassion” that underpins newer approaches to psychotherapy. The paper explores the origins of caring motives and the nature and biopsychosocial functions of caring-attachment behavior. These include providing a secure base (sources of protection, validation, encouragement and guidance) and safe haven (source of soothing and comfort) for offspring along with physiological regulating functions, which are also central for compassion focused therapy. Second, it suggests that it is the way recent human cognitive competencies give rise to different types of “mind awareness” and “knowing intentionality” that transform basic caring motives into potentials for compassion. While we can care for our gardens and treasured objects, the concept of compassion is only used for sentient beings who can “suffer.” As psychotherapy addresses mental suffering, cultivating the motives and competencies of compassion to self and others can be a central focus for psychotherapy.

## Introduction and Evolutionary Overview

### Background

There is a long history of philosophical and spiritual writings, highlighting the value of compassion as an antidote to suffering and anti-social behavior (Dalai Lama, 1995; Lampert, 2005; Ricard, 2015). However, it has only been in the last 30 years or so that we have seen substantial research on the neurophysiological, psychological, and social dimensions of compassion and compassion training (for reviews see Weng et al., 2013; Gilbert, 2017a; Seppälä et al., 2017; Stevens and Woodruff, 2018; Petrocchi and Cheli, 2019; Singer and Engert, 2019; Di Bello et al., 2020; Kim et al., 2020a). This work has been accompanied by the development of various forms of general compassion training (e.g., Jazaieri et al., 2013; Singer and Engert, 2019; Condon and Makransky, 2020) and cultivating compassion to address personal problems like self-criticism (Neff and Germer, 2017) and mental health issues (Germer and Siegel, 2012; Kirby and Gilbert, 2017). Among the latter, the most well-developed and evidence-based is *mindful self-compassion* of Neff and Germer (2017) to address self-criticism, and also *cognitively-based compassion training*, which combines the elements of cognitive therapy with Buddhist practices (Mascaro et al., 2017). This paper explores compassion focused therapy (CFT) rooted in an evolution informed, biopsychosocial approach to mental health problems and psychotherapy (Gilbert, 1984, 1992a,b, 1995, 2019). CFT is an integrative, multidisciplinary, process-based therapy that utilizes insights and wisdoms from many of the main schools of psychotherapy (Gilbert, 1989/2016, 2007a,b, 2019; Bell et al., 2020a,b; Fox et al., 2020) with increasing evidence of effectiveness (Craig et al., 2020; Fox et al., 2020). CFT was developed with and for people with mental health difficulties, particularly those who had not responded to other therapies, who had problems with self-criticism, shame, and trauma, often came from difficult backgrounds (Gilbert, 2000, 2010; Gilbert and Choden, 2013) and were fearful and/or distrustful of compassion from others and/or for self (Pauley and McPherson, 2010; Gilbert et al., 2011, 2014; Kirby et al., 2019). This paper is in two main sections. The first is an exploration of the evolution and processes of compassion and some of the key themes that underpin its application in psychotherapy. The second part explores the application of compassion to psychotherapy.

## Pursuing Compassion

Since there are different approaches to compassion and its application in psychotherapy (Gilbert, 2017b), this section explores the link between the evolution of caring and the emergence of compassion as a human motive and process. When trying to define compassion, we can start by noting that some approaches seek to identify particular core clusters of psychological processes and attributes (often suggested by the contemplative traditions) associated with it. These suggested core processes are then subjected to various forms of factor analysis (Strauss et al., 2016). Using this technique on a number of different approaches and measures, Strauss et al. (2016) concluded that:

A range of definitions from Buddhist and Western psychological perspectives were considered and five components of compassion were identified: recognition of suffering; understanding its universality; feeling sympathy, empathy, or concern for those who are suffering (which we describe as emotional resonance); tolerating the distress associated with the witnessing of suffering; and motivation to act or acting to alleviate the suffering. Each of these components has been articulated by several published definitions of compassion, although no single existing definition explicitly includes all five of them (p. 25)

While these are a commonly agreed set of processes for compassion, as with all such approaches, what we get out of a factor analysis very much depends on what we put into it. This is similar for the controversies in psychiatric diagnosis, which can use the same techniques with the same potential difficulties. Identifying core processes can help us to share conceptualizations but the exact phenomenon included in a cluster or factor can vary, sometimes quite considerably and in the clinical sciences be unreliable ways of identifying specific syndromes [see for example the controversies on one approach to self-compassion (Muris and Otgaar, 2020; Neff, 2020)]. As with psychiatric diagnosis, we can wonder if these are discrete states, traits, or if they are more fluid? Do we need all of the components for any particular act to be regarded as compassionate? Can compassion take place in the absence of (say) accurate empathy (see below), and is there a role for moral and rational compassion where we override what we actually feel (Loewenstein and Small, 2007)? Additionally, many of these qualities are dimensional – we can (say) have “degrees” of being moved by suffering. These dimensions will be affected by relational qualities such as emotional closeness (liking) versus seeing others as enemies (disliking) and trust. All of these attributes may be present, but a person may not actually follow through and act compassionately (Poulin, 2017). So, although statistical approaches have important roles to play in identifying core ways of “being compassionate,” to understand the complex processes of compassion itself requires other approaches too. For a recent major review of these issues (see Mascaro et al., 2020).

### The Importance of Understanding How and Why Care-Compassion Evolved

Understanding how and why caring and compassion evolved gives insight into a whole range of biopsychosocial processes (e.g., Gilbert, 1989/2016, 2005a, 2009a, 2017a; Depue and Morrone-Strupinsky, 2005; Goetz et al., 2010; Porges and Furman, 2011; Keltner et al., 2014; Brown and Brown, 2015, 2017; Carter et al., 2017; Porges, 2017; Petrocchi and Cheli, 2019). Recognizing that compassion can be understood as *an evolved strategy*, supporting survival and reproduction, *and* as a basic, personally experienced motivation that can be in conflict with other strategies and motivations (Gilbert, 2000), such as self-focused competitiveness, offers insight into its role in social behavior and mental states. Hence, rather than focusing on a clustering of “symptoms” or suggested “attributes,” the evolutionary approach seeks the origins of compassion in the evolution of caring motives and behavior, which then allows for the identification of the phylogenetic journey of the algorithms and physiological systems that make caring-compassion possible (Gilbert, 1989/2016, 2005a; Goetz et al., 2010; Carter et al., 2017; Porges, 2017; Uomini et al., 2020). This paper outlines a hierarchical, evolution informed, biopsychosocial approach to compassion which enables insights into how lack of care-compassion, particularly early in life, can underpin mental health problems and how cultivating compassion can operate as a psychotherapeutic process, and promote prosocial behavior (see also Weng et al., 2013; Seppälä, et al., 2017; Gilbert, 2020a). Evolutionary approaches address two key questions:

First is the study of its phylogeny:

1. Given that compassion is emergent from caring motivation, we can identify the evolutionary challenges of reproduction that gave rise to certain forms of caring for offspring behavior.
2. We can then explore the motives and algorithms that facilitate those parental, investing reproductive strategies and how they served as a template for other forms of caring and helpful behavior to evolve.
3. The evolution of motives and algorithms require physiological infrastructures to support them. In the case of caring and compassion, candidates include the hormones oxytocin and vasopressin and the methylated part of the parasympathetic nervous system called the vagus nerve and different neurophysiological circuits.
4. Over time, those algorithms recruit and possibly give rise to different types of complex competencies that include ways of reasoning, empathizing, and mindful awareness. Just as these can be recruited to advance any motive, they are utilized in the pursuit of compassion motives.
5. As a social mentality, compassion has a flow in that we can be compassionate to others, be open to the compassion from others and be self-compassionate. For the most part, each flow uses the same psychophysiology competencies.
  Second is the study of its ontogeny:
6. Once the basic algorithms with their physiological infrastructures are identified, it is possible to explore how, over the course on an individual’s life, these motives and algorithms are stimulated, recruited, and become incorporated into processes such as self-identity. In essence, we are looking at *the phenotype of the motive* and in this case the phenotype of caring to compassion.
7. We can explore how motives like caring can recruit different types of emotion and cognitive competencies to cope with different types of context.
8. Like other motives, caring and compassion have facilitators and inhibitors that can be both external and internal.
9. Understanding these processes means that we can then begin to develop contexts and interventions that are specifically aimed to stimulate different contributing processes to compassion, such as identified physiological systems, cognitive competencies and behavioral training, and practizing certain types of meditation. These create a menu of interventions for people with mental health problems; for example, some clients may need particular help with developing vagal tone, others with becoming more empathic, others with fears and distrust of compassion, and yet others with experiencing caring motivation itself and (maybe) to tone down narcissistic self-focus.
10. By understanding these processes, including how motives can conflict with each other (e.g., for pursuing competitive self-advantage *versus* caring and sharing), we can create compassion focused and guided psychotherapies, education, businesses, and politics.

#### Strategies and Motives

The basic challenges of life are survival and reproduction, and all living forms have three basic life tasks that give rise to three basic motives: (1) being motivated to avoid harm, injury, and loss; (2) being motivated to secure the resources necessary for survival and reproduction, including sexual access and infant caring; and (3) being able to tone down those motive systems when “resource satisfied” and “safe” to allow “rest and digest” (Gilbert, 1989/2016, 2009a, 2020a; Thayer et al., 2012; Petrocchi and Cheli, 2019; Workman et al., 2020). The bodily functions for rest and digest cannot proceed if the animal is under threat or is energized for resource seeking; thus, indicating co-regulating processes. The ability to downregulate physical activation through rest and digest has long-term impacts on illness vulnerability and mortality (Thayer et al., 2012). These basic life tasks promote what is called *fitness*; that is, success in passing genes for specific traits to subsequent generations. As noted below, different emotions are associated with these different life tasks and motives. Within those broad categories are a range of specific motives; for example, finding food and shelter, or in the social domain, competing with others, forming alliances, mating, and caring for offspring.

Different species evolve different (fitness promoting) strategies and ways of pursuing these motives. Primary motives require the organism to be alerted, orientated to, and respond to, certain kinds of stimuli/signal in specific ways so they can approach resources, but avoid threats and harms (Gilbert, 1989/2016, 1993; Panksepp, 1998). Motives also generate behaviors to *seek out* certain kinds of stimuli/signal. Hence, in order for a motive to operate, it needs *an algorithm* to guide it, to link stimulus and response. There is no point in being stimulus sensitive, interested, and orientated to (for example) food or sex if the animal does not know what to do. Lions are interested in antelopes as a food source, but if they do not have the foggiest idea of how to hunt or kill prey, they will starve. Evolving *algorithms* that enable motives to be enacted, therefore, have to have both elements. They begin as feature detectors that enable animals to identify and pay attention to (take an interest in) different kinds of stimuli in the environment, *and then* respond to those stimuli in appropriate ways. With brains that can learn, exact behaviors may depend on learning, such as lions learning how to hunt and kill and where the prey “hang out.” As noted later, the evolution of complex human cognitive competencies has introduced fundamental and profound new ways by which emotions and motives are triggered, pursued, and expressed.

Algorithms can be identified quite simply as stimulus-response dynamics of *if* A *then* do B. Nearly all processes in the universe operate on algorithms that are predetermined and give rise to the laws of physics and chemistry. One’s air-conditioning works on an algorithm of *if* the temperature goes above a certain level, then it turns itself on. If it goes below a certain level, it turns itself off. All that it needs is a feature detector (in this case) for temperature that links to the response function of the system. This is essentially the same way physiological systems are built. For example, the immune system operates such that *if* certain foreign agents are detected, *then* immune responses are stimulated. So basically, algorithms are what motives require to operate. Here are some examples:

➢ *if* a threat (e.g., predator) *then* activate arousal and run. This algorithm requires feature detectors for certain types of threat (this links to the preparedness hypothesis such that we are more likely to develop fears of snakes and spiders than electricity or cars which will kill more people). Once detected, it will then trigger physiological systems that enable the body to act to defend against the threat. Defenses can be active, as in running away, or inhibitory, as in freezing or depressive collapse.
➢ *if* food *then* activate approach behavior, salivate, and eat. This algorithm requires feature detectors for certain types of food, including internal awareness (hunger) of the need to eat, linked into physiological systems, such as digest once eaten, the body has systems (a gut) to digest and utilize food.
➢ *if* sexual opportunity *then* approach and engage in courting behavior and copulate. This algorithm requires feature detectors for certain types of sexual stimuli with physiological systems that prepare sexual organs for action. In some species, triggers are pheromones. For others, it is visual stimuli and sometimes linked to certain times of the year.
➢ *if* threatened by a more dominant other *then* escape, or if not possible, then display submissive behavior. This algorithm requires feature detectors for certain types of signal indicating a threatening, powerful, dominant other, and physiological systems that will switch on defensive behaviors, such as submissive hunkering down and eye gaze avoidance.

Now, we can come to the reproductive strategies that give rise to particular motives with particular algorithms for caring behavior:

➢ *if* signal of distress or need *then* engage in trying to alleviate it. This algorithm requires feature detectors for certain types of (distress/need) signals emanating from another (e.g., offspring). This requires identification of the offspring (kin vs. non-kin) and identification of the nature of distress or needs (e.g., rescue if in danger, feed if hungry, and thermoregulate if cold). Prevention can also be built into this to the extent that nesting, for example, will take place out of harm’s way (Geary, 2000).

Algorithms can become complex and branch into sets of interconnecting algorithms of “*if* A *then do* B, *but not if* in the presence of C,” or “*only if in* the presence of D.” What that means is that the system needs a feature detector for “C” and “D,” and then those algorithms can interact. Another typical example is *sequenced algorithms*, where there is a menu of options so that if one response does not work, another is triggered. For example, in most stress situations animals will first struggle to overcome a stressor but if that does not work then physiological systems automatically switch into a different response pattern which is often to go into helplessness or shutdown states (Overmier, 2002). Third, as noted below, the evolution of complex cognitive competencies has had profound effects on how these human algorithms, motives, and emotions work (Barrett, 2017; Gilbert, 2019).

#### Algorithms and Definitions

Before proceeding, we can combine ancient, motive and algorithmic ideas to help define care and compassion (Gilbert, 2017a). In the Mahayana Buddhist tradition (Dalai Lama, 1995) and the evolutionary tradition (Gilbert, 1989/2016, 2005a; Keltner et al., 2014), care and compassion are basic motives. Although CFT has defined care and its derivative “compassion” (see below) in slightly different ways over the years, we now try to stick closely to the basic “stimulus – response” algorithm of caring. Hence, *the stimulus* is some sign of suffering distress or core need that if not met creates suffering, which triggers a motivation and action to try to do something about them. As we will see, this algorithm is very ancient; the provision of care for the purpose of protecting, addressing needs, and supporting flourishing in offspring, can even be detected in fish (McGhee and Bell, 2014). Derived from an exploration of the evolution of caring behavior (Gilbert, 1989/2016, 2005a, 2009a; Goetz et al., 2010; Keltner et al., 2014; Cassidy and Shaver, 2016; Mayseless, 2016; Melis, 2018) consistent with the Mahayana Buddhist tradition (Dalai Lama, 1995), compassion can therefore be defined as a basic algorithm of “*sensitivity to suffering in self and others with a commitment to try to alleviate and prevent it*” (Gilbert, 2014). The intention and focus of care-compassion is clearly different from other motives, such as competitive self-interest, cooperating, or sexuality (Gilbert, 1989/2016). Importantly, however increased sensitivity to suffering by itself can be associated with increased distress and depression (Gilbert et al., 2019). Hence, it is what we do and how we manage these feelings that is crucial.

However each of those motives can be enacted compassionately making compassion a ‘higher order’ motive. One other element to note is that although compassion tends to be focused on alleviation and prevention of *suffering*, CFT, and indeed other approaches, are broader than that and focus on caring behavior which also has the intent of addressing needs and promoting development and flourishing. This is partly why Choden and I introduced the concept of *prevention*, into the definition because if needs are not addressed, or support for flourishing is not given, then suffering is likely to follow (Gilbert and Choden, 2013). In the Mahayana Buddhist tradition, one of the major antidotes to mental suffering is enlightenment; that is insight into the nature of mind partly because that prevents suffering. So, the actions and training for prevention are implicited in this approach.

#### Evolution of Different Caring Motives and Algorithms

Identifying the evolved algorithms for caring behavior is difficult. For example, one of the roots for compassion is from parental caring behavior. However, many species demonstrate different aspects of caring behavior. For example, some fish show guarding behavior and will chase off predators. McGhee and Bell (2014) studied three-spined sticklebacks, where fathers provide the care and protection. They note that:

During the approximately two weeks that fathers provide care, they defend their nest from predators, fan the nest with their pectoral fins to provide fresh oxygen to the embryos and once the embryos hatch, retrieve fry that stray from the nest. During this period, offspring rely on yolk reserves provisioned by their mother prior to fertilization. Fathers do not feed offspring, but there is evidence that offspring antipredator behaviour …., mate preference …. and morphology …. can be sensitive to the effects of fathers (p.2)

They go onto discuss how paternal caring influences traits such as anxiety in offspring that impact their survival and how paternal caring influences the epigenetics of their offspring. Indeed, it is now known that across many different species, the quality of parental caring impacts epigenetics and can attenuate or amplify vulnerabilities to threat sensitivity (Cowan et al., 2016; Kumsta, 2019). What is also crucial here is that there are a number of *different behaviors* that constitute caring which may be regulated through different algorithms and physiological systems. Hence, a “father” fish may be good at (say) rescuing straying offspring, but less good at fanning the nest. As noted later, compassion can also be seen as made up of multiple different sensitivities and behaviors depending on context. People may be good at certain aspects of compassion, but not others.

Care of offspring is not the only source for the evolution of caring. Kessler (2020) explores the evolution of what she calls “health-care,” caring for sick individuals, and highlights that many species care for their sick and injured. Kessler refers to the work of Frank et al. (2018), who noted that termite hunting ants (*Megaponera Analis*) are prone to injury such as losing legs, but are often carried back to the nest by nest mates, where their chances of recovery are 80% compared to 10% of those who are not. Once healed, these ants can return to the group tasks. Kessler also highlights a range of evolved caring behaviors (e.g., grooming) whose function appears to be parasite and infection control. It is common that solutions that work in one species can independently evolve in other species. This is true for caring (Spikins, 2015, 2017; Uomini et al., 2020).

Identifying *specific* motives to *care for the sick* is interesting on two fronts. First, at times, this appears to be a specific type of caring and compassion, where individuals can be very motivated to care for the sick or those in danger, but not invest so much in their own families or close relationships. They spend more time in the hospital on duty or working for charities; public and social rather than intimate forms of caring (Gilbert, 1989/2016). To put this another way, some individuals may be specifically sensitive to signals of “sickness or danger” in others, or “committed to a cause” if there is a “danger to the physical body,” but less care and compassion orientated or competent when it is mental states or intimate relating.

Another “life challenge” that may have supported the evolution of caring and compassion is the degree to which certain traits are attractive to others and make individuals desirable as mates and allies (Goetz et al., 2010). It has been known for a while that females prefer altruistic over non-altruistic males, particularly for long term partners and to some degree “heroism” that could be used to protect (Margana et al., 2019). When it comes to forming friendships and reciprocal relationships that can be mutually beneficial, signals of caring, altruism, and trustworthiness also play a key role. Hence, evolving traits for caring is also evolving traits that have beneficial effects on one’s potential to be chosen as a sexual mate or cooperative ally (Goetz et al., 2010). Note, too, that context can play a big role for what and to whom we are compassionate. For example, in groups of fighting men, individuals who are fearful and somewhat avoidant maybe shamed and shunned, whereas fearfulness and avoidance is a central focus for compassionate psychotherapy. In general then, caring has evolved from different selective pressures with different feature detectors that distinguish different types of distress and need signals requiring different types of intervention (e.g., rescue, comfort, or feed).

#### Altruism, Sacrifice, and Compassion

Another evolutionary angle on the origins of compassion is through the concept of altruism. The evolution of altruism is typically seen to be driven by two processes: (1) kin-based, where caring behavior has a payoff for one’s genetic representation in the next generation and (2) reciprocal, where helping behavior will result in being helped in the future (Buss, 2015; Colqhoun et al., 2020). In a review, Preston (2013) offered the following definitions for altruism, which are very close to the concept of compassion.

Altruistic responding is defined as any form of helping that applies when the giver is motivated to assist a specific target after perceiving their distress or need “…..” Altruistic responding implies an active behavioural response initiated by the perception of need, which is differentiated from cooperative, diffuse, or unintentional forms of altruism that likely derive from other evolutionary and mechanistic origins… Altruistic responding further narrows these classifications to only include cases where the motivation to respond is fomented by direct or indirect perception of the other’s distress or need… This excludes cases that emerged later in time or include diverse processes, such as cooperation or helping influenced by strategic goals, social norms, display rules, or mate signaling (p. 1307; italics added).

For Preston (2013), the origins of altruistic responding are in the evolution of detecting and responding to (retrieving/rescuing) distress calls in infants – coming to their aid. Like CFT, she identifies passive and active forms of caring. Passive forms are providing soothing and comforting, whereas active forms are specific behaviors designed to rescue or alleviate distress in infants and require motor activation. Unlike compassion, this definition of altruism excludes the concepts of sharing or acts that focus on the flourishing and well-being of others or “general caring.” Helping people that does not have a cost or can actually benefit oneself in the long term is questionable as to how altruistic-compassionate it is (Colqhoun et al., 2020; Miller et al., 2020). Clearly, the ultimate benefit of kin-caring is the flourishing of one’s genes in the next generation, sometimes called *inclusive fitness*. Given that helping behavior is energy expensive, then one would predict far more of it will be given to kin relations and immediate reciprocating others than distal strangers. For the most part, this is exactly what the research demonstrates, that we lavish huge resources on our own children and kin even though we know many other thousands of children around the world will die every day as a result of lack of food, clean water, and simple vaccinations (Colqhoun et al., 2020). And, we are highly focused on our own groups even to the point of physiologically responding to pain differently if it is a member of one’s own group or a different group (Hein et al., 2010). Importantly, Richins et al. (2019) suggested that this empathy inhibition can occur if groups are in competition, but is less noted if they are not. This is an important finding because it indicates that one motive system, such as competitiveness, can change a range of processes such as empathic engagement.

Undoubtedly, humans are prepared to make sacrifices of their own lives to save others, and even strangers, as witnessed in many situations of the rescue services or medical staff working on viral infections around the world including of course Ebola and COVID-19. It is not entirely clear, however, when that kind of altruistic behavior evolved (Kessler, 2020). Nor is it clear when we develop the capacity to make sacrifices of giving up our own resources at a cost to ourselves. For example, although helpful behavior has been observed repeatedly in young children, most research has been when there has been no cost to them for helping. Green et al. (2018) investigated how helpful young children would be if there were a cost. They found that children would help a hand puppet achieve a goal of completing a task (e.g., puzzle) if there was no cost to them, but helping fell significantly when they had to give up something to help the puppet. Even when the puppet made appeals and was clearly distressed, the child still would not give up their own resources or rewards to help the distressed puppet; and even sometimes when they were clearly distressed at their own refusing to help behavior (Kirby personal communication). In a different paradigm, Miller et al., 2020 did find that four to six year olds were prepared to give up things important to them to help others and this was related to heart rate variability and also the child’s experience of maternal care and love. In other words, children growing up in loving and caring households are more likely to care and share with others.

#### Social Mentalities and Caring

Co-evolution is the way species evolve because of their interactions. For example, prey will evolve attributes (camouflage and escape speed) that enable them to escape predators. This drives predators to become better camouflage detectors and faster pursuers. Cleaner fish evolve in relationship to bigger fish; viruses and bacteria evolve ways to exploit the defenses of hosts. *Social* co-evolution, however, is different because of its focus on communication and social signaling as information flows between one or more individuals that have direct physiological impacts (Gilbert 2017c). For example, if infants evolve motives and competencies for distress calling, but parents do not evolve the motives and competencies to notice (be stimulus sensitive and “interested”) and respond in specific ways, evolution cannot progress along that dimension. Equally, if mothers evolved capacities for caring and soothing, but infants do not evolve capacities to be receptive and physiologically influenced by those signals, then again, evolution cannot proceed. Hence the relationship (e.g., kin vs. non-kin; friend vs. enemy) triggers the algorithm. Change the relationship and a signal of distress may not activate caring behavior (Mascaro et al., 2020). The quality and type of the relationship textures the processing of social signals. Hence, we can distinguish between non-social motives that do not require the evolution of complex social signaling processing and those that do. Avoiding heights or water (if one cannot swim) and finding food and building shelters are not social. Motives for competing, sex, and caring can *only be successful where there is a contributing (not always willing) partner* forming interpersonal, reciprocally dynamic dances. Hence, social mentalities are role focused, recruit different algorithms, physiological systems and cognitive competencies to engage in *different interpersonal dances* in the co-creation of different roles (Gilbert, 1989/2016, 2005b, 2017c, 2020a; Hermanto et al., 2017; Mullen and O’Reilly, 2018; Brasini et al., 2020).

The co-evolution of the motives and competencies for forming *many* different types of social role (e.g., dominant-subordinate, sexual, caring, and sharing) has profoundly influenced the evolution of multiple physiological and structural processes. Lockwood et al. (2020) reviewed considerable evidence looking at the specificity of social behaviors and brain function which is very supportive of social mentality theory. In the case of caring, researchers have drawn attention to adaptations to the central and autonomic nervous systems that facilitated the co-evolution of care providing and care-seeking forms of relating (Gilbert, 1989/2016, 2005a; Porges, 2007, 2017; Thayer et al., 2012; Brown and Brown, 2015, 2017; Carter et al., 2017). Not only has the evolution of caring behavior brought modifications to the central (Depue and Morrone-Strupinsky, 2005) and autonomic nervous system (Porges, 2017), for another example of the importance of specificities of processes underpinning social mentalities, consider Carter et al. (2009) discussing the evolution of the middle ear:

the middle ear permits detection of high-frequency airborne sounds (i.e., sounds in the frequency of human voice). Even when the acoustic environment is dominated by low-frequency sounds, the development of the mammalian middle ear also was critical in the evolutionary history of sociality because it allowed the mother to eat, nurse and listen to conspecific vocalisations at the same time (p.172).

In essence then, the middle ear in humans was driven (partly) by co-evolution for caring, rooted in social communication, and provided one essential competency for the evolution of speech. It is because social motives depend upon co-evolution and complex reciprocal, dynamic communication processes, they have been called social mentalities (Gilbert, 1989/2016, 2005b, 2017c). The importance of decoding (from others) and sending signals to others (conspecifics) in complex interpersonal dances is hugely important because it gives rise to competencies for early forms of empathy and mind-reading (Lockwood et al., 2020; Luyten et al., 2020). In addition, these interactional “dances” have profound physiological impacts, including on epigenetic profiles (Cowan et al., 2016). Hence, many mammals can distinguish between signals of caring, signals of threat, signals of sexual interest, and signals of submission, etc., and will have evolved different responses for each. More specifically, they can distinguish between very different types of distress and suffering that require very different types of intervention and distinguish between different targets (e.g. kin vs non-kin).

#### Caring and the Body

The physiological infrastructures underpinning caring and compassion have been subjected to considerable research over the last 20 years (for reviews see Porges and Furman, 2011; Keltner et al., 2014; Brown and Brown, 2015, 2017; Mayseless, 2016; Gilbert, 2017a; Seppälä et al., 2017; Stevens and Woodruff, 2018). We know, for example, that the hormones oxytocin and vasopressin played vital roles in the evolution of caring behavior not only for infants, but also pair bonding (Carter et al., 2017). Variations in this gene may also link to variations in compassion and prosocial behavior (e.g., Tost et al., 2010; Marsh, 2019). Changes to the autonomic nervous system, particularly the myelination of the 10th cranial nerve of the parasympathetic system which evolved to become the vagus nerve, played a significant role in the regulation of threat and the soothing qualities of connectedness (Porges, 2007, 2017; Stellar and Keltner, 2017; Petrocchi and Cheli, 2019). It now looks as if the parasympathetic *rest and digest system*, which regulates (sympathetic) threat and drive states, was incorporated into close relating enabling the signals emanating from a parent to have soothing vagal-mediated qualities on an infant (Porges and Furman, 2011; Porges, 2017). Indeed, different physical interactions of the parent to the infant (e.g., touching, holding, stroking, voice tone, and feeding and processes of intersubjectivity) have considerable but different physiological regulating effects (Hofer, 1994; Porges and Furman, 2011; Siegel, 2012; Cozolino, 2014; Schore, 2019). Supportive of mentality theory there is now considerable evidence that mother and baby can synchronise processes within their autonomic and central nervous systems. These synchronies can be thought of as symphonies and dances between their physiological systems that profoundly impact phenotypes for subsequent prosocial behavior and mental health (Lunkenheimer, et al., 2015; Nguyen, et al., 2020). Furthermore, there is good evidence that the vagus plays a major role in prosocial behavior and caring and compassion in general (Keltner et al., 2014; Petrocchi and Cheli, 2019; Di Bello et al., 2020).

### Caring Functions and Attachment Processes

As noted above, offspring caring has been identified in multiple species including fish, avian and mammalian, and offspring are epigenetically affected by the quality of the care they receive (Cowan et al., 2016; Uomini et al., 2020). In many species, the interactions between the infant and primary care giver pertain to feeding, thermoregulation, rescuing, and comforting. These effect the maturation of different *physiological systems*, such as autonomic nervous system, neurochemistry, and immune function (Hofer, 1984, 1994; Brown and Brown, 2017). As a psychiatrist interested in human psychological processes and the development of mental health problems, Bowlby (1969, 1973, 1980) offered a functional analysis of the impact of parental care-giving/receiving relationships on *psychosocial systems* and development. He described three core functions associated with what he called attachment (see Cassidy and Shaver, 2016; Music, 2017 for reviews). As noted below, each of these three functions are very important functions of compassion too. When developing a compassionate mind or turning to a compassionate other they are central. These are:

1. *Proximity seeking/maintenance* relates to feature detectors for access and availability to a caring other, staying close to each other for protection and care, and if lost to find and rescue, and if hungry feed. Many physiological systems are maturing in the context of this close, interpersonal connectedness that can be disrupted with ruptures to that closeness (Hofer, 1994; Wang, 2005; Siegel, 2012). Hence, part of proximity seeking and maintenance is to support maturational interpersonal dances. These are impacting physiological levels, brain development, and epigenetics (Cozolino, 2014; Cowan et al., 2016), and a range of psychological competencies (e.g., empathy) are all developing in this context of closeness (Siegel, 2012; Cozolino, 2014). Indeed, throughout life, the way we maintain our proximity to each other, touch each other, smile, and joke with each other, show affiliation, speak with each other (even on the phone and Zoom), help each other, have fundamental impacts on a range of physiological systems (Cozolino, 2014; Petrocchi and Cheli, 2019). We are a physiological, co-regulating species.
2. The provision of a *secure base* provides an environment free from threat, where infants can explore and learn; the parent acts as the “eyes for threat” and “guards the child” allowing the child to focus attention on exploration, play, and learning. As a secure base, the parent “mediates” the world for the infant, titrating threat exposure, guiding discoveries, and promoting skills development with developmental needs for learning (Gilbert, 1989/2016; Cassidy and Shaver, 2016). This is similar to aspects of a working therapeutic relationship (Holmes and Slade, 2017). Along with guidance, a secure base is also a source of encouragement and inspiration. In the context of a secure base and caring parent, the child experiences playful, joyful, exciting, and affectionate interactions (Schore, 2019). They learn that they exist *positively in the minds of others* and that others love and like them, and hence develop (positive) internal working models of self in relationship to others (Gilbert, 1989/2016, 1992a; Cassidy and Shaver, 2016; Music, 2017). Out of the complex melange of varied and multipurpose interactions comes our ability to trust others. The more we experience others as providing a secure base for us, the more we trust them, and the more we trust them, the more they are able to provide a secure base of encouragement and guidance (Fonagy et al., 2017). As we grow, our secure base extends to networks of peers and other individuals (Hrdy, 2009; Narvaez, 2017, 2020). Importantly too, a secure base sets clear boundaries for participants in the relationship enabling predictability, but also maintaining respectfulness and care for participating individuals. A secure base *is not* overly permissive or allows disrespectful or destructive behaviors, or gives in to every “want.” A child who has not learned age appropriate respect for others or impulse control is problematic. This is true in social networks in general. Part of creating secure bases in communities is to have a very clear system of laws, and encouraging citizen respectfulness as a central social behavior.
3. A *safe haven* is where the parent acts as a soothing and emotion regulating other, particularly but not only when the infant-child is distressed. Humans are biologically setup to expect and need emotion regulation to come from the outside and they are physiologically prepared to respond to that – indeed throughout life the care of others can sooth us. Whereas a secure base is partly about stimulating, encouraging, and inspiring, as well as approving and admiring, a safe haven in some ways is the opposite. This is the ability to be soothing and calming often in the context of high arousal, usually threat, but sometimes overexcitement or impulsiveness. Evidence suggests that a lot of safe haven functions operate through the rest and digest system and, in particular, the myelinated vagus nerve, a view developed by Porges (for a review see Porges, 2017; Stellar and Keltner, 2017). In his classic terry cloth (no food) vs. wire (with food), mother surrogate studies in primates, Harry Harlow showed that it was *physical contact* rather than feeding that was the crucial stimulus for soothing (for review see Harlow and Mears, 1979). So soothing stimuli and secure base stimuli are different, and therapists need to guide clients to that awareness so that they can distinguish between them. In other words, when distressed, some aspects of caring and compassion will be about encouraging, supporting, validating, and guiding into and not away from areas of difficulty, whereas others will be about soothing and containing physical distress. It is incorrect to see caring and compassion in CFT as only linked to safe haven function and not secure base function.

Attachment theorists highlight that if one has experienced these in early life, they become internalized such that we can provide them for ourselves (Cassidy and Shaver, 2016). Hence under stress, we remember to “internally proximity seek;” that is we can be sensitive to our distress with a capacity to tune into and generate an inner sense of soothing (safe base) and self-reassurance and encouragement (secure base). So, vital are these inputs to the infant and child, that failure to receive them, or worse experiences of threat and abuse in early life, reset strategic orientation at a phenotypic level (Cowan et al., 2016; Del Giudice, 2016; Kumsta, 2019; Slavich, 2020). The consequences of insecure attachment history have been well-documented and are known to be major sources of mental health problems and anti-social behavior later in life (for reviews see Music, 2017; Lippard and Nemeroff, 2020). In people with mental health difficulties, these inner abilities to provide themselves a sense of secure base, reassurance, and safe haven as in grounding and soothing are often weak or absent. In their place are forms of hostile self-criticism which amplify (sometimes drastically), rather than dampen threat processing (Gilbert and Irons, 2005; Castilho et al., 2017). In other words, when we are distressed, suffer setbacks, or disappointments rather than having an internal secure base and safe haven, we activate the threat system through harsh self-criticism (Gilbert and Irons, 2005). For those with anti-social problems, the focus is on stealing exploiting or harming others.

Increasingly, many therapists have been influenced by attachment theory and understand the importance of creating a secure base and safe haven (Gilbert, 1989/2016; Rothschild, 2000; Van der Kolk, 2015; Holmes and Slade, 2017). Indeed, for some traumatized clients, this is an essential therapeutic endeavor. Importantly, however, is to recognize that feeling safe is different from safety and that both are important, and that in the early days efforts to help people feel safe, for example, trying to stimulate the vagus nerve, might actually make them feel more anxious. See below on fears, blocks, and resistances. It is crucial therefore to distinguish safety from safeness as the latter is more tricky than the former.

### Safety Versus Safeness: A Crucial Distinction

#### Safety

Many forms of mental health difficulties are linked to the way individuals are subjected to, monitor, process, and cope with threats (Gray, 1987; Gilbert, 1989/2016, 1993; Rachman, 1990). This puts *threat management* center ground to most therapy (Rachman, 1990). CFT distinguishes two quite different basic systems involved with threat regulation and explorative behavior (Gilbert, 1989/2016, 1993, 2000, 2009a). Gray (1987) described a threat focused, behavioral inhibition system (BIS) that is triggered by signals indicating punishment and reduction in rewards (losses) and unexpected or novel stimuli. General levels of threat arousal can influence the degree of threat monitoring. These can activate *the responses* of the BIS which are: increased arousal, focused attention, and behavioral inhibition. Part of what anti-anxiety drugs and therapies are designed to do is to tone down sensitivity in the BIS. In contrast, is a behavioral activation system (BAS) linked to drive and resource seeking behaviors. It is triggered by signals of reward/resource, an absence of punishment and when activated individuals show higher levels of goal seeking behaviors and positive emotions (see Carver, 2004 for a pros and cons discussion). Various therapies seek to build these too.

**Figure 1:**
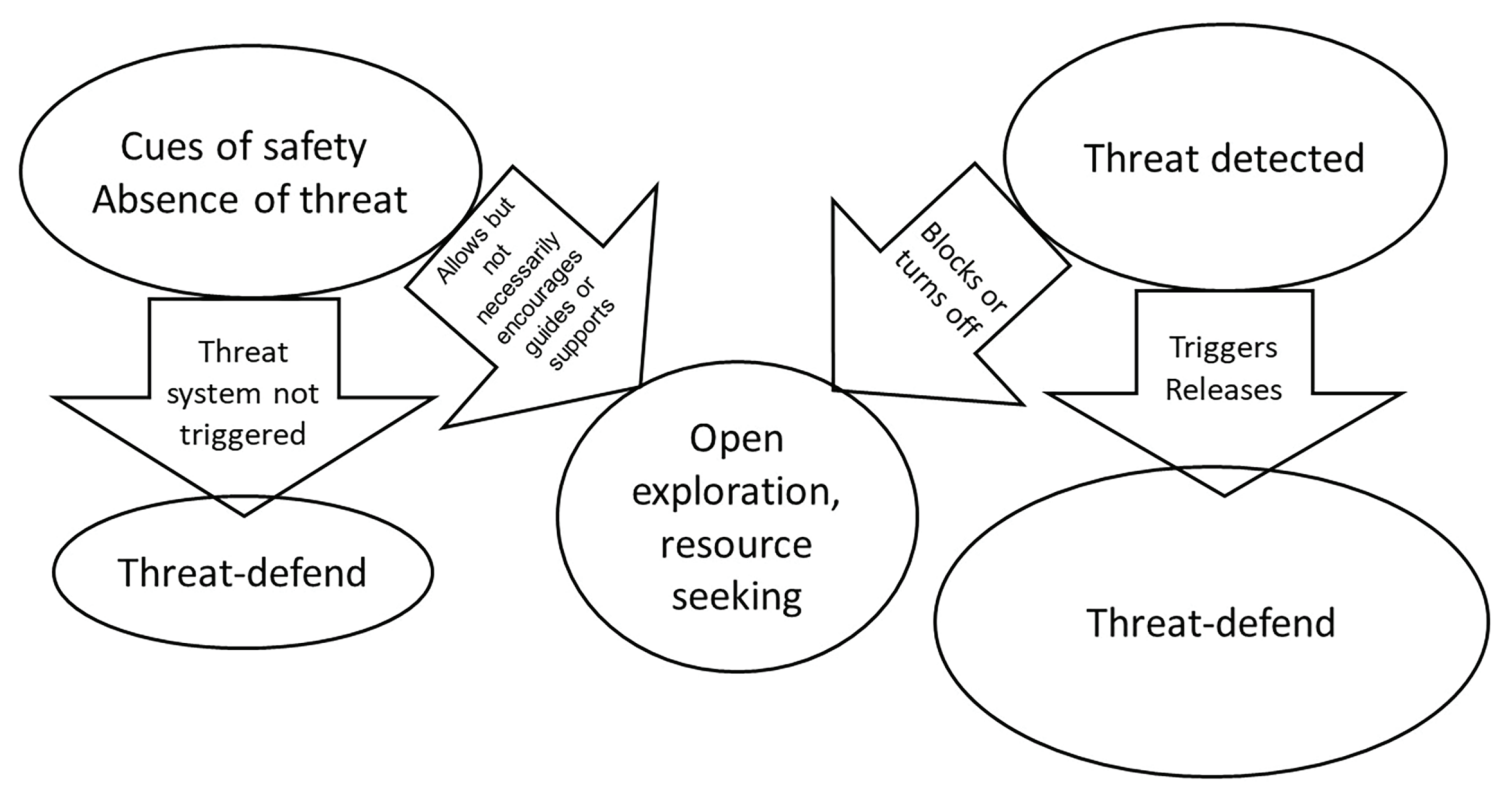
Safety and threat processing © Paul Gilbert.

Riskind et al. (2012) highlighted that the degree of threat people experience is related to the degree to which they see threat as “looming,” in terms of different types of distance, such as physical and temporal distance and probability of arrival. Individuals also make calculations on the degree of danger and harms and if they can be offset, prepared for, or avoided (Mobbs et al., 2009). All of these can be related to *safety seeking* and *harm prevention or damage limitation*. Attentional sensitivity and coping behaviors operate through the physiology of the threat system such as the amygdala, hypothalamic-pituitary-adrenal axis, and sympathetic nervous system (Gray, 1987; Panksepp, 1998). So, we have a sense of safety when the threat system is not picking up on threats, and therefore, is not being stimulated (although is ticking over in the background). This is sometimes called the *smoke detector* principle (Nesse, 2019), although that does not capture the dimensionality of threat (a little vs. a lot). This is depicted in Figure 1.

In everyday life, it is important that we pay attention to safety behaviors, such as putting on our car seat belt, COVID mask, or taking the vaccine. The focus on safety *is prevention of harm*. While many animals will need to balance threat against opportunity (seek for food in predator environments), the safety seeking system can be constantly “braking” or interrupting explorative behavior to stay vigilant. Watch feeding animals in the wild. Some have argued that interhemispheric differences in the brain evolved to enable animals to engage in activities like feeding while at the same time attending to and keeping “an eye open for threats” (McGilchrist, 2018). McGilchrist argues that the constant conflict between threat vigilance and securing resources was the source of hemispheric differentiation and lot of emotional difficulty, partly because the two hemispheres process information quite differently with quite different functions. Indeed, studies of people who have had their corpus callosum-lesioned, because of epilepsy, have shown that these two parts of the brain can make quite different decisions (see also McGilchrist, 2018; Schore, 2019). Clients who get stuck in the threat monitoring system can live (aspects of) their lives with elevated vigilance “to looming threat” (Riskind et al., 2012).

Figure 1 is reasonably self-explanatory. It simply shows that in the absence of threat signals the threat system remains low key, but an absence of threat will not necessarily promote exploration, play or positive social engagement. A detected threat will activate the threat system and then will suppress other behaviors not related to dealing with threat. There are obvious individual differences in the degree to which people monitor for both ‘the presence and absence of threat’. There are of course many other ways in which we can have a sense of safety and regulate the BIS. The BIS might have become oversensitized, due to difficult childhood experiences or be regulated through learning skills we have confidence in. Imagine how you might feel if someone threatens you, but you are a Bruce Lee super karate expert. Having an internal model that we can cope with certain threats obviously influences our monitoring (Mobbs et al., 2009). CFT and other therapies try to help people shift out of inappropriate vigilant and safety seeking motives, by developing confidence to appropriately assess threat, and the courage to tolerate and manage threat (Rachman, 1990; Riskind et al., 2012). This then enables a shift in the BIS-BAS balance and be able to pursue behaviors and build relationships that are more enjoyable, but also engage the rest and digest system more functionally. But the awareness of threat itself is not sufficient for us to understand threat regulation.

#### Safeness

In 1989, I suggested another dimension for threat regulation that I called the safety system (Gilbert, 1989/2016, p. 88). Using the same reasoning as Gray, of identifying the algorithm in terms of its triggers and responses, I suggested the system was triggered by cues of no threat *and also* cues (as in attachment theory) of supportive others. However, one did not need to have an *attachment relationship* with others, but to see others as supportive and helpful. Early background would lay the foundations for openness to these social communicative signals and stimuli. These signals and stimuli did two things. They: (1) deactivated the threat-defense system and (2) promoted reward/resource seeking and affiliative behavior. The creation of friendly relationships then became self-reinforcing such that affiliative relationships activated safety and the positive rewards/emotions of feeling safe increased the orientation toward affiliation. Prosocial behavior, therefore, was a way of creating interpersonal relationships that were mutually pleasurable and beneficial. Sending signals of safety and safeness to each other have major physiological impacts (Gilbert, 2000; Porges, 2007; Petrocchi and Cheli, 2019).

I had opportunities to discuss the issue of social versus non-social stimuli with Gray and how that related to his BAS and he offered some very insightful concepts. I used them in Gilbert (1993). By 1993, the distinction between safety and safeness was becoming more apparent, and so in 1993, I referred to the system as a safety/safeness system, but still lumping the two functions (monitoring absence of threat and monitoring presence of support cues) together. It was the late primatologist Chance (1988), who became a colleague and who on reading my paper on safety and threat (Gilbert, 1993) advised me that I needed to more clearly separate these functions. We discussed how there is big differences between threat vigilance (absence of threat) and feeling safe. These differences were likely to be physiological as well. He used the example of group behavior. In any group which is competitive or where there is a hierarchy that is potentially punitive (common in many primates), individuals focus on safety by ensuring they do not behave in ways that stimulate conflict with those more powerful. The group *looks* stable, but this is basically what he called an *agonic mode*, stability through threat and submissive wariness and defense. Families or groups with threatening or bullying parents/bosses can be like this. Levels of stress arousal tend to be high. However, when relationships are friendly and people can trust each other to not be attacking but supportive, the whole group settles into a very *different structure of attention* and shared physiological arousal; here, there is much more openness and sharing. He called this the *hedonic mode*. *Safeness* was when the environment is caring, supportive, helpful and friendly and creates these states of open attention. One of the transitions from primate to hunter gatherer in humans was partly moving away from aggressive hierarchies into hedonic mode type group relating (Boehm, 1999; Ryan, 2019). Ten years later a fascinating, natural event reflected Michael’s view. Robert Sapolsky and team (Sapolsky and Share, 2004) had been studying a group of baboons when some of the dominant males ate from a poisoned rubbish dump and died. This left the group with relatively less aggressive males and females and the group then settled into a much more relaxed state noted in patterns of grooming and general levels of arousal (lower stress) which was maintained even with other males migrating in.

I remain fascinated in how to integrate attachment theory and safeness concept into compassion concepts. Sometime later, I discovered that Steven Porges had explored the functions of the vagus nerve and outline the evolution of what he called a social engagement system, which is very similar to the social safeness system. Articulating very carefully the role of the myelinated vagus nerve, it became known as *polyvagal theory* (Porges, 2007, 2017). In CFT, we tend to stick with the concept of social safeness rather than engagement, because one can socially engage for all kinds of reasons (Kelly et al., 2012; Armstrong et al., 2020). However, there is a lot of overlap between the two approaches. So, this brings us back to the evolution of how to create a secure base. This is partly created by recognizing boundaries but also by experiencing the friendliness and helpfulness of those around you. This promotes exploration because one is *not vigilant to threat*; and one’s exploratory behavior is not constantly being interrupted by having to check out on threat. When one feels safe, one has relaxed attention, can explore and play. Social relating are positively rewarding and enticing. This also provides the basis for integrative thinking and learning. Clearly, this is a state you want to create in therapy.

We can explore the concept of safeness and how different it is from safety with attachment theory. Bowlby (1969, 1973) suggested that attachment systems are evolved to work on threat in a different way. The mother acts as *a signal of safeness* that the child moves toward rather than away from. She is a source of food comfort and protection and when the infant avails themselves of these resources, this stimulates positive reward centers, not threat ones. As a result of different types of repeated interactions such as physical closeness, feeding, thermoregulation, touch/comfort (Hofer, 1984, 1994) the mother is stimulating oxytocin-endorphin-parasympathetic circuits (Depue and Morrone-Strupinsky, 2005; Lunkenheimer et al., 2018). Over a number of years, Porges (2017) has delineated the special role of the vagus nerve in these functions. What this means is that in her presence, these systems are active and will be suppressing threat system processing. As long as the infant has access to her (or primary care giver) then the infant has access to food, comfort, thermoregulation, protection, guidance, etc. Crucially for CFT, these lay down internal working models such that we can provide them for ourselves; in essence that is the basis of *self-compassion to function like a good parent* to ourselves and stimulate these oxytocin-endorphin-parasympathetic links when we need to.

**Figure 2:**
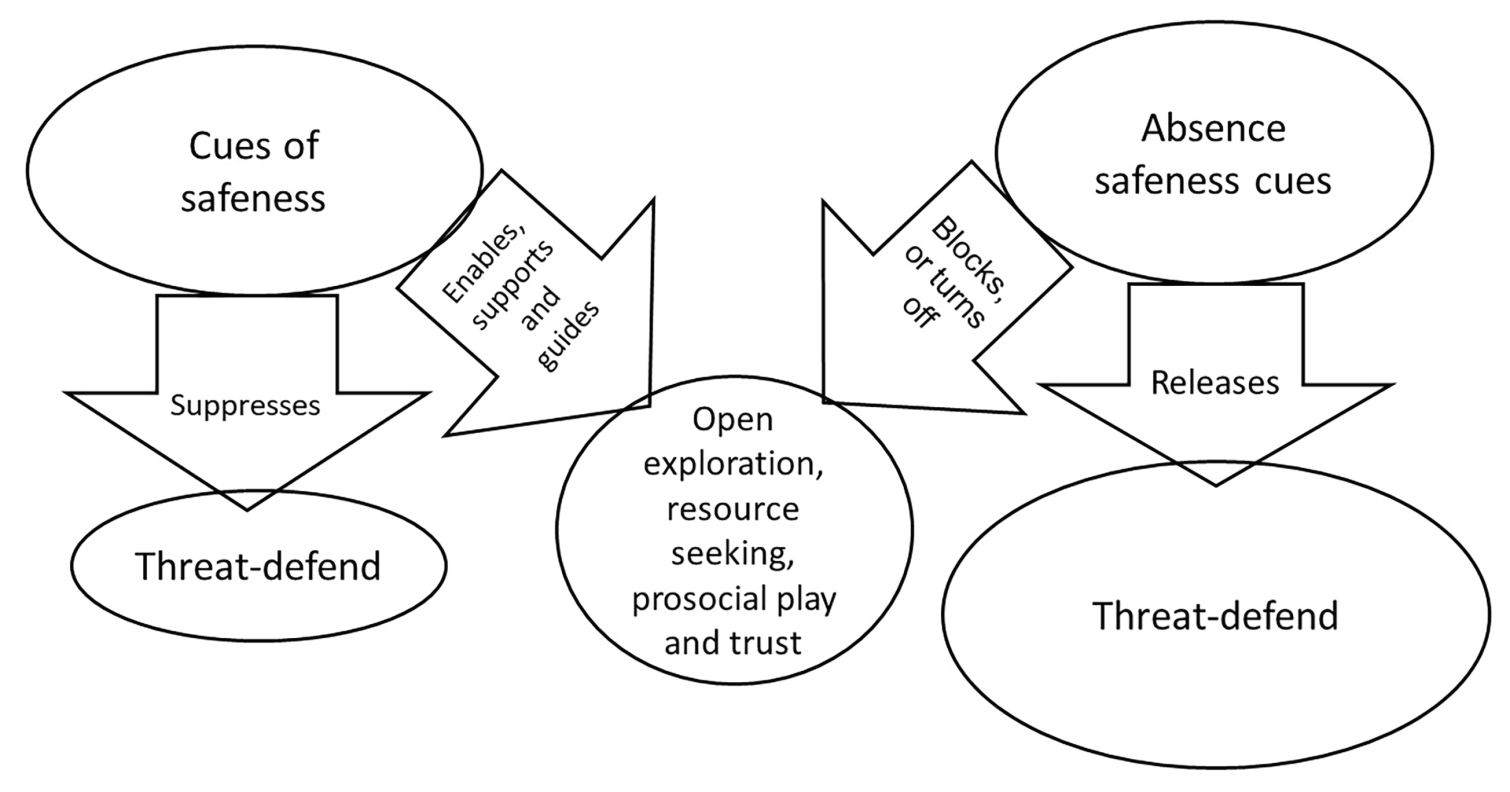
Safeness and threat processing © Paul Gilbert.

A number of early classic experiments showed that when a reliable parent is present, children will show positive affect, curiosity, play, and engage in explorative behavior (see Cassidy and Shaver, 2016 for reviews), what Chance (1988) called a hedonic mode of interaction. In addition, the parent may support, encourage, and guide these behaviors. This is clearly not the case if it is simply a safety vs. threat regulation processing issue; affiliative play is not a part of that. If the parent becomes unavailable or separated (e.g., leaves a room), there may be no actual threat in the room, yet this immediately *releases threat system processing* and the infant’s motive is then searching for the safe-providing object (mother; see Siegel, 2012; Cozolino, 2014; Cassidy and Shaver, 2016; Schore, 2019 for reviews). In other words, the presence of safeness signals (a trusted parent) *suppresses threat system processing*, and in so doing, can bring other systems online such as explorative play which in turn can build affiliative relationships. Hence with their removal (mother goes out), this *releases the threat system* from its inhibition and orientates the child to now pay attention to the (evolutionary important) task of threat vigilance and seeking the return of the safe object. These processes are depicted in Figure 2.

The key element implicited in the mode approach of Chance (1988), attachment theory (Bowlby, 1969), and social mentality theory (Gilbert, 1989/2016, 1992b, 2000) is the process by which the presence or absence of “safeness” signals facilitate, release from inhibition, or suppress threat processing. Individuals who are prone to anxiety and stress, therefore, are not just experiencing *facilitators* of threat processing, but also the lack of *inhibitors* of threat processing such as access to caring and support from others or self.

#### Relaxed and Safe Are Different

This issue showed up in another way. Wanting to see if we could develop a scale that would tap different types of positive emotion, particularly those linked to excitement and achieving versus linked to rest and digest and “chilling out,” Gilbert et al. (2008) developed a short self-report questionnaire to tap these different dimensions of positive emotion. While excited/energized types of positive emotion formed a separate factor to that of soothing and calming emotions, what we had not anticipated is that relaxed, positive affect is different to “feeling safe” and contentment positive affect. Importantly, the latter was more powerfully linked to mental health issues. This again suggests that experiences of safeness overlaps with, but are not the same as, rest and digest, but rather link to a sense of social safeness and connectedness (Kelly et al., 2012). This was also found by Matos et al. (2018). In other words, when people feel safe, particularly as provided by social contexts, they may feel content which quells excessive resource seeking and threat focused behaviors (Cordaro et al., 2016). Armstrong et al. (2020) found that feelings of safeness could be seen as a separate type of positive emotion that can blend with playfulness or relaxation.

When people feel “socially safe” they are not necessarily in calm, relaxed, or low arousal states, but could be playful, curious, and enjoying social events like parties and further developing relationships with friends and lovers. Keltner et al. (2018) also review some of the subtleties and differences in the autonomic nervous system relating to subtle variations in positive emotions such as awe, excitement, love, and contentment. Indeed, we could argue that many aspects of joyful social relating from sexual encounters through to going to parties, sharing jokes are examples of creating and experiencing social safeness. It is these dimensions of caring that are so essential for creating these interpersonal experiences that regulate our states in mind. Knowing that people care about you, and will address your suffering and distress if needed, helps to create a sense of safeness and builds trust and closeness.

#### Priming Social Safeness

There is considerable research showing that safeness signals downregulate threat. For example, Heinrichs et al. (2003) showed that when subjected to certain types of social stress, a person’s level of cortisol is influenced not only by oxytocin, but also by the presence of a friend. In a priming experiment by Norman et al. (2015), participants were shown “48 pictures depicting caring behavior and individuals enjoying close attachment relationships, such as hugging loved ones” (p. 833). Control subjects were shown pictures of household objects. Being primed with sharing and caring relationships had the effect of attenuating amygdala responses to threat stimuli from facial expressions and dot probe. Kumashiro and Sedikides (2005) showed that people were much more likely to engage with, and be curious about, negative feedback on a task if they had been previously primed with bringing to mind close positive relationships. Hornstein and Eisenberger (2018) did not distinguish safeness from safety, but nonetheless show that what they are calling safety signals, and what I am calling safeness signals, can have powerful impacts on the acquisition of fear and the extinction of fear. Recently, Gillath and Karantzas (2019) provided an extensive overview of the forms and effects of attachment priming. They found that priming attachment security [or what we refer to here as “caring other(s) and social safeness”] studies have used different methodologies, including subliminally and supraliminally exposing people to security words, such as affection, love, and support, names of attachment figures, and pictures of people hugging a child, thus recalling memories of being loved and supported. Priming people in attachment security (or social safeness; Kelly et al., 2012) impacts positively on how people process a range of potentially threat-based scenarios, including tendencies to make them more pro-social. Kelly and Dupasquier (2016) found that student population experiences of social safeness and sense of connectedness mediated the relationship between recalling parental warmth, and the degree to which individuals were fearful of being self-compassionate or being open to receiving compassion from others. It would appear that for us to be able to use compassion, it helps if we have experienced it in our primary relationships.

Signals indicating safeness can also relate to one’s community (e.g., feeling we live in a safe-helpful neighborhood or with safe and supportive neighbors). Keep in mind that these are not just communities that are low threat, but also communities providing a sense of social connection and mutual support; sometimes also seen as social capital. Social safeness is a powerful predictor of well-being, even more than positive and negative effects (Kelly et al., 2012). CFT distinguishes between social safeness and non-social safeness in the sense that being wealthy, and having money easily available, makes the world feel safer than if we are poor. Some forms of an over-reliance on seeking social safeness, however, can be problematic as in those individuals who have what are called dependency problems and who become anxious without access to helpful others; or those who use wealth to feel safe, but want more and more.

Recently, Slavich (2020) wrote extensively on what he called *social safety theory* linking a whole range of physiological systems and their interactions including: the immune system, hypothalamic pituitary adrenal stress system, the vagus nerve, the lymphatic system, and frontal cortex as very sensitive to an individual’s safety. Indeed, it is another facet of the importance of how various phenotypes, developed in safe versus threatening social environments, can channel individuals to quite different strategic orientations in life. However, a distinction between safety and safeness would help to clarify these processes. What are the physiological effects of being in a relatively low threat environment and how might these be different in a socially enriching, fun, caring, and supportive environment? Although calling it safety rather than safeness, Slavich has offered major new insights into the importance of social safeness and social connectedness (Kelly et al., 2012; Armstrong et al., 2020) on the regulation of multiple physiological systems.

To cut a long story short then, when looked at physiologically, removal of a threat versus the presence of safeness stimuli regulate threat processing *via* different physiological systems. Especially important is that some degree of a secure base and sense of safe cues of safeness and helpfulness build courage. We cannot build courage by trying to remove ourselves from the source of threat (avoidance). Hence, whereas the absence or removal of a threat stimulus will *deactivate* threat processing, the presence of affiliative and social safeness signals *stimulate* the oxytocin-opiate circuits, vagus, and have soothing and/or encouraging qualities (Depue and Morrone-Strupinsky, 2005; Carter et al., 2017). Note, too, how research on “lovingkindness” has powerful and direct effects on threat processing systems. Weng et al. (2018) showed that such meditation practices changed the sensitivity in amygdala to threat signals. It is partly because clients use avoidance as a way of regulating threat, rather than developing courage to tolerate and engage with it, that problems escalate for them (Rachman, 1990). Repeatedly then, CFT tries to help clients build the psychological and physiological infrastructures for creating an internal sense of safeness, secure base, and safe haven. Bratt et al. (2020) explored the experiences of adolescent girls with CFT. One of the central experiences for them was “gaining courage to see and accept oneself.”

On a neural level, secure versus insecure attachment styles have been shown to moderate the degree to which individuals engage in self-criticism during fMRI. Specifically, at greater levels of an amygdala response during self-criticism (i.e., threat), individuals higher on secure attachment measures have greater neural response within the visual cortex, as a potential marker of engagement in imagery than those with lower scores (Kim et al., 2020a). Importantly, this effect was not observed for individuals with higher avoidant attachment scores. This may indicate that an avoidant attachment style is associated with a greater tendency to disengage from the threat of self-criticism, whereas secure attachment is associated with the ability to tolerate the threat of self-criticism. This may explain why an avoidant attachment style is associated with criticizing others rather than criticizing self (Mikulincer and Shaver, 2016). In addition, people who see themselves as superior tend to blame others rather than themselves for their difficulties (Gilbert and Miles, 2000).

As we have come to understand these processes, therapies are now targeting them in a kind of neuropsychophysiotherapy (Gilbert, 2000, 2009a, 2010; Cozolino, 2017; Fiskum, 2019). For example, compassion training has been shown to produce changes in the autonomic nervous system as measured by heart rate variability (Matos et al., 2017; Di Bello et al., 2020; Kim et al., 2020c; Steffen et al., 2020) in the immune system (Pace et al., 2009) and in various cortical areas (Weng et al., 2013, 2018; Singer and Engert, 2019; Kim et al., 2020b). Useful too are compassion scales developed to measure both positive levels of compassion engagement and action (Gilbert et al., 2017), and fears of compassion (Gilbert et al., 2014; Kim et al., 2020b) are also sensitive to detecting changes in HRV (Di Bello et al., 2020). Put simply, research is finding that the higher one is in compassion engagement and action the better their HRV, but the more fearful they are of compassion the lower their HRV. In addition, Singer and colleagues (e.g., Singer and Engert, 2019) have shown that specific types of interventions such as mindfulness, empathy, and compassion, stimulate different neurophysiological systems.

## From Evolved Functions to a Compassionate Focused Psychotherapy

One of the early observations that inspired CFT was finding that while working with cognitive behavior therapy (CBT), clients could sometimes generate helpful thoughts to counteract negative, self-accusatory, and attacking ones, but these were not always helpful. When I asked a particularly severely depressed client to speak out her “helpful” thoughts *as she actually heard* them in her mind, her emotional tone was aggressive and contemptuous. Helping her begin to develop a compassion motivation and genuine caring emotional textures to her depression, life tragedies and internal dialogues proved a lot more difficult than I had anticipated. I began to explore the same issues with other clients and sure enough they could generate coping thoughts with helpful content but not with any sense of a compassionate motive or emotional texture. Many clients found that even talking to themselves in a compassionate, sensitive, and caring way is very difficult. Pauley and MacPherson (2010) explored the reasons they found self-compassion difficult. In addition, being open and moved by the compassion of others was also difficult to believe, feel, or trust. This led to an exploration of how and why developing motivation, intention, and emotional textures of caring and compassion can be so difficult for some yet be such a powerful antidote for mental health problems. It also reaffirmed the centrality of that motivation for any intervention (Gilbert, 1989/2016, 2000). Part of the explanation involved understanding the function and psychophysiological mechanisms underpinning care motives, compassion and their fears, blocks, and resistances (Kirby et al., 2019). For the most part then, CFT was developed with the more complex and chronic mental health problems and was guided by many of the experiences and recommendations for development that clients offered on their journeys that have improved therapy (Gilbert and Procter, 2006). The therapist uses psychoeducation so that the client has clear insight into *why* they are doing the practices, particularly in terms of developing different competencies, and brain and body states they might not have had a chance to develop in childhood.

Having explored the evolution of care and the specific functions of care on psychosocial development (which is to provide a secure base and safe haven which in turn sets up an internal regulation of threat by the cultivation of a safeness system or process), the rest of this paper will explore how these insights can be used to guide and support psychotherapy. While many CBT therapies focus on helping people deal with threats fairly directly and often helpfully, this is mostly by working with the threat system itself either through exposure or cognitive change. CFT, however, seeks to work with basic motivational systems and, in particular, how to create inner capacities for feeling supported, and hence able to activate “safeness processing” and to develop mutually supportive, prosocial, and caring relationships, and to live ethically.

This paper does not have space to outline the various interventions and processes of training for developing compassionate mind states and competencies (Gilbert and Choden, 2013; Gilbert and Simos, in press). Simply to say that there are a range of practices and interventions such as breathing practices that stimulate the vagus, a range of different visualizations and meditations, exploration of compassionate reasoning, and compassionate behavior, some of which are guided by understanding the physiological underpinnings of caring compassion. Particularly, important is for clients to begin to understand how to create an inner sense of a secure base and safe haven that counteracts (among other things) shame and self-criticism which they can turn into when distressed and also utilize as a source of encouragement and guidance. These are related to what we call the compassionate self, mind and the compassionate image.

### Mapping the Mind

To explore how compassion fits into psychotherapy, we need to explore briefly the different dimensions of functioning that give rise to mental suffering and how care and compassion motivation reorganizes these processes. It is extremely important that therapists do not see working with compassion as a simple a “add on,” but understand how it is used to change the organization of the processes we are going to discuss; ultimately, we need to be able to have long-term physiological impacts (Cozolino, 2017; Schore, 2019; Steffen et al., 2020) and turn states into traits (Goleman and Davidson, 2017).

**Figure 3:**
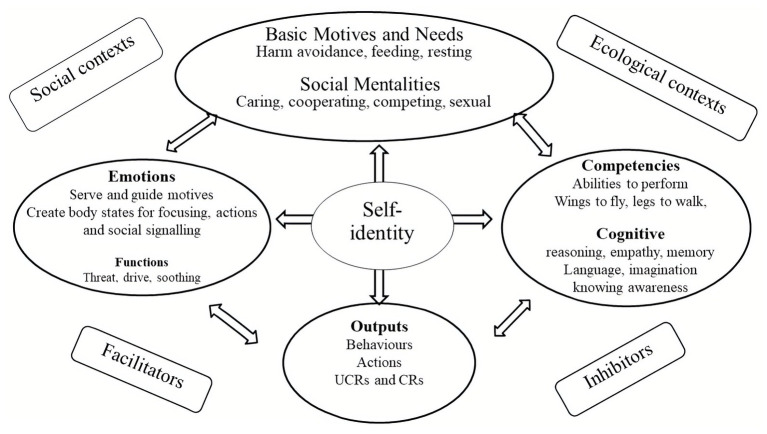
Mapping the mind. Adapted from Gilbert (2019).

People seeking psychotherapy for distressed states of mind present with many different types of difficulty. Psychotherapists require some overview of the domains of functioning that will become a focus for intervention. In addition, articulating different domains of function enables description of how these functions operate in a motivation such as compassion. Taking the basic psychological science approach, CFT focuses on four main domains: motives, emotions, competencies, and behaviors (Gilbert, 1989/2016, 2019). These are depicted in Figure 3.

#### Basic Motives and Needs

From an evolutionary point of view motives run the show. All life forms are faced by three major life tasks which provide the basis for motives. These are: (1) motives to avoid harm, injury, and loss; (2) motives to acquire social and non-social resources promoting survival and reproduction; and (3) motives to rest and digest when not defending against threats or needing to pursue resources. As noted below, CFT refers to social motives, to create certain types of role relationships as *social mentalities*. This is partly because they have to co-evolve and create complex interpersonal dances; e.g., between carer and cared for, sexual partners, co-operators, dominant and subordinate, leader and led. Motives are also linked to needs such that we obviously need to eat and (early in life) to be cared for, have a sense of belonging and be able to compete for our share of resources and status (Gilbert, 1989/2016; Neel et al., 2016). If these relationships are compromised, if we feel excluded or rejected, unloved or unwanted, marginalized, oppressed and subordinated, we will suffer. These types of unmet needs or overdeveloped pursuits (e.g., for power) are often a focus in psychotherapy. One of the crucial insights that we have been aware of, even before Freud, is that the mind is full of conflicting motivations, emotions, and beliefs which can be very difficult to understand or regulate (Gilbert, 2000). CFT helps clients to recognize that behind any decision, there can be many different motives. For example there are often motives for, getting married or divorced or changing jobs. Therapists can plot these out in a ‘mind map’.

In many situations, we are often confronted with opportunities to be caring, sharing or self-focused, acquiring, and achieving. Indeed, the more competitive we see the world, and our need to keep up, the less compassion we may have (Piff et al., 2018) and the more vulnerable to mental health problems (Curran and Hill, 2019; Gilbert, 2020b). This is partly because competing for resources immediately opens up the potential of threat from competitors, but also failing. Importantly, whatever the source of our suffering and mental anguish, if we can activate a care focused and compassion motivation system, this will organize our minds in such a way to make it easier to deal with our suffering. Using the definition above, this means that as we shift into care and compassion motivation, we are oriented to be sensitive to ours and other peoples’ suffering; try to turn into it, not away from it, understand it, and then work out the best way to be helpful. This is clearly different from trying to avoid being sensitive to the suffering of self and others, or trying to suppress it, or becoming callous and indifferent. There is increasing evidence that social pressures to be competitive also increase callousness (Piff et al., 2018).

#### Emotions

One of the major reasons people come to therapy is because of what they feel, that is problems with their emotions and their moods (Greenberg, 2003). While there are many varieties, textures, and approaches to emotions, taking an evolutionary functional approach, Nesse (2019) suggests that “Emotions are specialized states that adjust physiology, cognition, subjective experience, facial expressions, and behavior in ways that increase the ability to meet the adaptive challenges of situations that have reoccurred over the evolutionary history of a species” (p. 53). Basically, emotions serve motives and prepare the body for certain types of action and cognitive focus. CFT uses an evolutionary *clustering of emotions by function* approach. The main functions are as supports for primary motives noted above: (1) threat and harm avoidance, (2) resource seeking and acquiring, and (3) rest and digest and soothing (Gilbert, 2005a, 2009a, 2014). Hence, we have emotions that will help us take actions to defend ourselves (anger, anxiety, and disgust); emotions to help us to seek out and repeat behaviors that bring in resources and rewards (joys, fun, and various pleasures), and emotions that allow us to settle, rest, and recuperate (safeness and peaceful contented).

**Figure 4:**
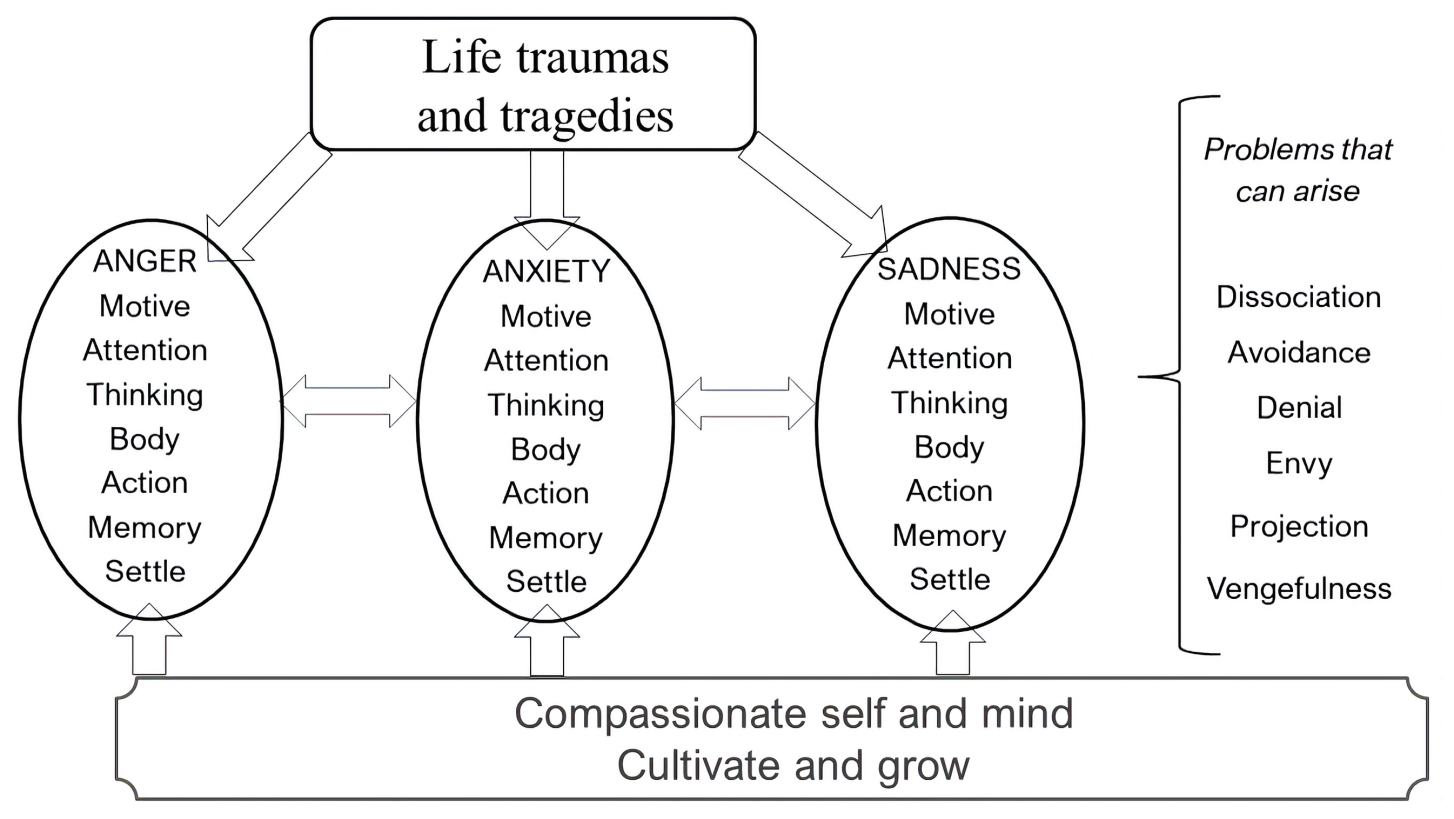
Multiple responses to life traumas and tragedies © Paul Gilbert.

Central to CFT is the recognition that the brain is a pattern generator and can generate multiple biopsychosocial responses to the same stimulus/event. This is particularly true for threat and harm events. For example, imagine working with abuse trauma, or with major life tragedies such as being diagnosed with cancer or losing a loved partner. CFT suggests that we are commonly hit by what we call “the big three:” anger/rage, fear/anxiety, and sadness. Disgust is also important, but less common in the “usual” traumas and tragedies. Because our experiences are *about patterns of functions and processes*, each of these will have their own motives, attention profiles, ways of thinking, body patterns, action impulses, memories, and ways of settling. They can function like “mini-selves” in a way. CFT explores each of these in turn. This is an exercise called *multiple selves* which helps clients to understand that we are a collection of multiple possibilities not a unitary self (Gilbert, 2000, 2020b; Bell et al., 2020a,b; see Figure 4).

Version of Figure 4 can be shared with clients or drawn out with clients. So, for example, imagine the therapist working with a threat like cancer or other loss or trauma and says that: “*when we are struck by these types of life events, we can often have a lot of very difficult and complex emotions. There can be a part of us that's very angry about what has happened to us, ’why me, why now’ for example. Then there may be another part of us that is very frightened of the consequences and implications for the future. But there can also be a part of us that's very sad and grieving, because the tragedy or trauma has caused considerable losses in our life.*” The therapist then invites clients to reflect on that and see if that resonates with them. CFT suggests that clients benefit from understanding and exploring their multiplicity. This practice and guided discovery offer opportunities to *differentiate* between the different emotions by being guided to focus on one at a time. We can then explore *their relationship* by asking “what does angry self think about anxious self? What does anxious self think about angry self”? and so on. This helps clients to recognize that our emotions can often be in conflict making things even more difficult. Sometimes, people recognize that behind their rage can be great sadness or vice versa.

When the client begins to understand this multiplicity, they can then shift into a compassionate mind state and work compassionately *with each emotion individually*. Over time, clients begin to *recognize their multiplicity*, which is also extremely beneficial for mindfulness. Having differentiated these different emotions, people are much more able to recognize them as they are arising. This makes bringing compassion to them easier. Commonly too clients can be invited to literally step in and become that emotion, like an actor stepping into a role, become it, and in so doing clients sometimes have different insights (Bell et al., 2020a,b). Note that on the right-hand side of Figure 4, there are a number of problems that can arise when people struggle with differentiating their emotions and/or are ashamed or frighten to acknowledge or experience. Them the point is that we can hold both the emotions and our reactions (such as our envy or vengefulness), compassionately; that is without shaming and blaming, but simply experiencing, noticing, and finding a compassionate way to work with these basic emotions.

This type of “multiple pattern analysis” can also be very useful for working with dilemmas. For example, suppose a person is in a dilemma of whether to leave or stay in a relationship. The therapist can invite them to take up the dilemma in one of two (or more) chairs and explore the motives, attention, thinking, feeling in the body, behavioral impulses, and memories and “best outcome.” When the client has experienced each aspect of the dilemma, they are then invited to take up a position or sit in a “compassion chair,” go through a number of the practices such as breathing and compassion focusing of the mind to bring online the compassionate mind (e.g., with vagal activation; Gilbert and Choden, 2013; Porges, 2017) and then, from that position and mind state, reflect on the dilemma. They can then reflect on what they have learned about it, including that most dilemmas involve some kind of major loss. Indeed, it can often be discovered that it is the loss we are less prepared to suffer that guides decision-making. Sometimes, dilemmas are because we have avoided addressing issues. For example, a client is not sure whether to stay or leave their job because the boss is somewhat bullying. This work may help them recognize that before deciding whether to stay or go, because they like the actual job, learning to be assertive, or seek the support of others might be the first steps.

##### Positive Emotions

It has been known for some time that the ability to recruit positive emotions to help cope with life setbacks provides important sources of resilience and “bounce back” (Fredrickson et al., 2000). Hence, this would be important for compassion as it shows that being able to stimulate positive emotion helps to address suffering. However, rather than treating positive emotion as a single dimension, CFT highlights the importance of distinguishing between two types and functions of positive affect, suggested by the work of Depue and Morrone-Strupinsky (2005). They distinguish affiliation from agency and sociability. Agency and sociability are linked to control and achievement seeking, social dominance, and the (threat focused) avoidance of rejection and isolation. Warm and affiliative interactions, however, are linked to social connectedness and safeness as conferred by the presence and support of others. Affiliative social relationships calm participants, alter pain thresholds, help immune and digestive systems, and operate *via* the oxytocin-opiate system and vagus nerve (Depue and Morrone-Strupinsky, 2005). So, we must distinguish between positive emotions that are about building and broadening, from those that are about contentment and slowing.

When it comes to positive emotions, Hanson (2020) highlights the fact that our minds can be like “teflon for positive emotion and velcro for negative” because we have negativity biases (Baumeister et al., 2001). Increasing positive affect is often *via* behavioral interventions and mindfulness techniques (see below in behaviors). In CFT, we have exercises that specifically focus on: (1) the activating excitement and pleasurable and (2) the more settling and calming effects of contentment. Importantly, however, some clients have fears of either or both (Gilbert et al., 2014). Indeed, one of the reasons people fear compassion is because they are fearful of positive affect in general; for some, it can seem alien or a potential source of threat.

#### Competencies

To perform any function, organisms need competencies. For example, birds need wings to fly and also brains to navigate them in the air. Flying is neither a motive nor an emotion, but any emotion or motive can recruit competencies. Hence, a bird may fly to find food, to escape predators, and to seek out sexual partners. Competencies that distinguish humans from other animals are our cognitive ones. The early roots of many of our cognitive, “problem solving mind” and intelligent competencies can be traced back to early life forms and are clearly present in other species. For example, cephalopods and invertebrates like octopus (Amodio, 2019), various avian species (Marino, 2017), and primates (Byrne, 1995) have a variety of complex cognitive competencies that allow them to solve their life challenges. Nonetheless, humans have evolved a set of at least three types of cognitive competencies that clearly set us apart from other species. These include:

Our capacities for reasoning, systemic thinking, planning, symbol, and language that underpin problem solving and science (Byrne, 1995; Suddendorf and Whitten, 2001; Suddendorf, 2018; Workman et al., 2020). Because we can think in time, we can think of what to do and what to train in today to help us tomorrow or next year. Language and writing enable us to store and accumulate knowledge, and develop and pass wisdom *through the generations*. These competencies are often targeted in the cognitive therapies that help people look at the way in which they are reasoning, drawing inferences, making attributions, and so forth.

A second major competency relates to forms of *empathy and mind awareness* (Decety and Ickes, 2009; Baron-Cohen, 2011), which many see as essential to compassion. These competencies are very important to become role sensitive and competent in certain roles. For example, individuals who are able to tune into the emotions, motives, and needs of others are more likely to be successful when it comes to any social motive such as mating or building alliances, outwitting competitors, and of course, caring. These competencies are often seen to support some forms of compassion and are a focus in psychotherapy; in other words, the empathic skill of the therapist, and the training in empathy the therapist gives to the client, impacts outcome (Luyten et al., 2020). However, empathy is not equally distributed and can be inhibited to out groups (Hein et al., 2010) and competitor groups (Richins et al., 2019).

Nonetheless, to what degree do we need empathic *accuracy* to behave compassionately? To what extent can men understand what it is like to have a baby or a menstrual cycle? People of diverse ethnicity argue that it is difficult for (say) white middle-class people to understand their cultural, racist experiences in white-dominated societies. Indeed, many argue that without certain shared experiences, it is difficult to “fully” empathize. Clinicians have reported that before they themselves became seriously depressed, they did not fully understand what it was like to suffer from depression, even though they had been treating it for years. People who have lost those they have loved sometimes acknowledge that they did not recognize what a deep state of yearning grief is really like until it happened to them. Lewis (1961) begins his observation of his grief over the death of his wife of just a few short years, that he had not realized how much fear he would experience.

On another level, to what extent do we actually understand the experience of animals? Tragically, the denial of suffering in animals has underwritten horrendous cruelty to them, as in the whaling industry and factory farming. Loewenstein and Small (2007) argue that there are many routes to compassionate and ethical behavior, and that relying on empathy and sympathy may not be sufficient to ensure we deliver moral and compassionate behavior in the areas that we need to. There are times then when being empathic means that we are aware it is difficult to be empathic and we *need to listen and imagine*, recognizing our empathic limits. Nevertheless, even if we cannot be empathic to the details of suffering, we can still wish to address suffering skillfully.

Bloom (2017a,b) goes further and indicates how, where, and why empathy can even lead to harmful outcomes. If one is Machiavellian or wants to be deceptive, empathic skills will help. Weiss and Zaki (2017) conclude their overview of empathy-building interventions with a sentiment long expressed in CFT by saying; “Although these approaches have been successful, intervention should also benefit from adopting a complementary, motive-based approach that targets the underlying forces governing empathy (p. 213).” This has been a prominent feature of CFT for some years and is why it is important to distinguish between a motive and a competency. Empathy as a *competency* can be recruited into the service of any social motive. So again, we come back to the crucial role of motivation in how competencies are used.

An increasing number of therapies recognize that empathy training of different kinds is necessary, both personally and as a therapist (Weiss and Zaki, 2017), with the mentalizing therapies being at the forefront of such in psychotherapy (Fonagy and Adshead, 2012; Luyten et al., 2020). However, like mindfulness, empathy training by itself, without understanding the motives, utilizing these competencies may not be put to good uses. As Eyal et al. (2018) notes, becoming more empathic can take us more deeply into pain, which will need to be tolerated; otherwise, people will turn away from it (Eisenberg et al., 2015). Hence, the key role of courage. One reason for shutting down empathy is because it can open up overwhelming feelings or fear. One colleague mentioned that “I never want to think about people being tortured because it just overwhelms and frightens me.” Sometimes, therapists think they are being empathic but are projecting and imagining *themselves* in the situation, not their client in that situation. Projection can be a difficulty. Imagine I am empathizing with a marathon runner. If I imagine *myself* as a marathon runner, it would be very different to imagining *being* this other super fit human being dedicated to train with a love of running. I am not either! There is a difference between imagining oneself in a situation and imagining the client in that situation. We often make assumptions that people will feel what we feel and that can be a failure of empathy. Hence, at the same time, empathy requires us not to blur our boundaries and get lost in the experience with the other.

Therapists can have genuine difficulty in empathizing with people who have caused harm, particularly sexual abusers, for example. Nevertheless, this is important if we are going to be able to work therapeutically with them. It is, in fact, an act of *courageous compassion* to be prepared to move into the dark side of another persons’ mind and hold a compassionate position based on the wisdom that we all just find ourselves here and nobody chooses the brain they end up with (Gilbert, 2010; Ashbach et al., 2020). Hence, to some extent, therapeutic empathy means the ability to own and be able to move around in one’s own shadow and dark side, visit painful, and frightening areas, while always maintaining independence of the self, and not blurring self-other boundaries.

A third type of competency relates to “awareness of awareness” a type of *consciousness of consciousness*. Some might argue that this type of awareness is not cognitive as such, more of a faculty of mind. Holding that on one side for the moment, the fact is that we can think a thought or have an emotion or desire *and consciously know* that we are having a thought and emotion. The faculty of knowingness underpins the ability to be mindful. *Knowing awareness and knowing intentionality* have been game changers. While a lion can obviously intend to stalk and kill to eat, it does not knowingly do so. It cannot suddenly decide to become a vegetarian or wake up in the morning to go training to get fitter; as far as we know it cannot reflect on its own actions and decide to hunt in a different way tomorrow or ask questions about the meaning of life. *Knowing intentionality* allied to other forms of reasoning intelligence have changed the way in which basic motives are expressed and developed for good and for bad (Gilbert, 2018). In the last 20 years, various forms of mindfulness training have been incorporated into different schools of psychotherapy (Siegel, 2010) and to help with stress (Bohlmeijer et al., 2010; Gu et al., 2015).

It helps to see these as separate competencies because they are not always related. Some individuals can be brilliant scientists, but poor at empathy or mindfulness. Others may have degrees of empathy but appear to lack caring motivation, as in psychopathic traits (Baron-Cohen, 2011). Some individuals can be very empathic, but will not win Nobel prizes, and some people can be very mindful, but not necessarily empathic, compassionate, or that scientifically orientated. Indeed Singer and Engert (2019) have shown that different “mindfulness” trainings impact the brain in different ways. Hence, in CFT, we might be helping people to reason compassionately or practice compassion focused body work or develop empathy or distress tolerance and develop mindfulness as a skill that supports compassion; and more besides. Clearly, these skills will advance processes of mind awareness and differentiation between given motives and emotions.

Brought together then, CFT highlights competencies that help us to develop the courage to engage with suffering rather than avoid, runaway or dissociate from it, and those competencies that enable wise action. As a useful heuristic, these have been presented as interacting circular processes, because they are not linear and independent (see Figure 6 below) to be discussed later.

#### Behaviors

A fourth dimension is the behaviors that we manifest, and these can be regulated in a range of different ways. For example, behaviors can be directly tied to ecologically important stimuli called unconditioned responses. Many basic behaviors such as the so called four Fs, fight, flight, feeding, and sexual behavior are the action parts of the algorithms of the motives. Behavior therapy is, of course, well-known to focus on helping people engage in specific actions and behaviors and to rehearse desired behaviors. We learn to drive by driving, not by reading about or meditating on driving. When we want to act compassionately, we may have to learn and practice specific behaviors.

There are many ways *guided behaviors* can help people to become more mindful and aware of sensory pleasures and “positive” emotions (Hanson, 2020). Behavior therapy for depression focuses on intentionally and deliberately, increasing potentially rewardable behaviors. Sometimes, it is helping clients recognize they can explore behavior experiments to help them to “slow down and learn how to utilize their rest and digest system” to facilitate experiences of contentment and being “good or sufficient enough” without feeling failures or let downs. Some clients have lived trying to please others and have a very poorly developed sense of their own desires and preferences. The technique of guided behaviors can be used to (for example) explore the value of playfulness (which sometimes feels very strange to some clients) “what would your excited, playful, contented, self etc., be motivated to pay attention to, think or do? How could you bring that more into your life; why might that be helpful; what might worry you about developing your ability to be playful or fun loving?” Or you might explore different kinds of behaviors and their meaning. One depressed lady who had lost her joy in cooking was invited to cook something to bring to therapy and be mindful about all the different elements in her cooking, the choice, the ingredients, textures, the smells, the colors, the anticipation of my enjoyment (or fear of not) of her food and so on. This is mindfulness in actions. Cooking could also be seen as a kind of play, to learn to focus on the process, *not the outcome*. For some clients, helping them literally learn “how to play,” can be important particularly if they come from a neglectful, rather empty emotional landscapes. For compassion, this also clearly overlaps with concepts such as the 8-fold pathway (Gilbert and Choden, 2013).

### From Caring to Compassion: The Knowing Mind

**Figure 5:**
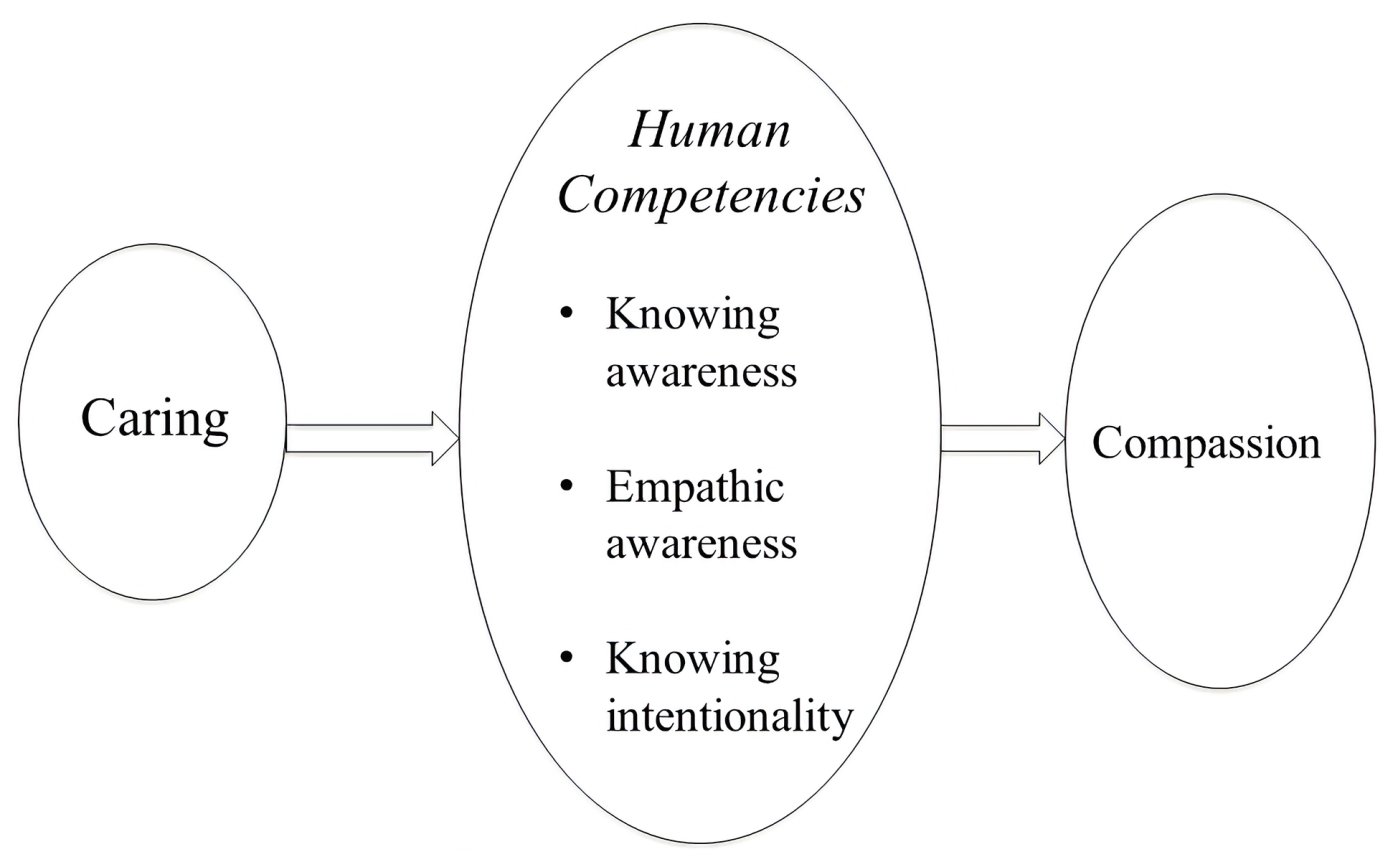
From caring to compassion. Adapted from Gilbert (2018).

Up until this point, we have been assuming that compassion and caring are pretty identical, but in fact, that is not quite the case. To a large extent compassion depends on our cognitive competencies. There are many explanations for the origins of these types of human competencies. One is that it was social processes, and in particularly, caring processes that drove them and social intelligence (Dunbar, 2009, 2014; Spikins, 2015; Uomini et al., 2020). Another is that of Machiavellian intelligence (Byrne, 1995). Since competencies can be used by any motive, it is quite likely that different motives combined to generate the ones we have today. Whatever their source, it is clear that these competencies are indeed game changes and impact on the activation and expression of all of our motives, including caring. Clearly, many species care for their offspring, but we would not call all caring compassionate. Just as Goodall (1990) pointed out that animals are not really capable of cruelty because they do not *intentionally set out to cause suffering* and hence cannot be regarded as cruel (see Gilbert, 2005a), we have compassion when we *intentionally and knowingly* choose to address suffering. Hence, both cruelty and compassion are possible because we can “know” what we are doing, we can *understand the consequences* of our behavior on the physical and mental states of another. CFT suggests, therefore, that it is when caring motives are orientated through our human cognitive competencies that caring becomes compassion. In the Buddhist traditions too, this is why mindfulness and compassion training often go together (Gilbert and Choden, 2013; Ricard, 2015). This is depicted in Figure 5.

#### Compassion and Sentients

Another dimension where caring and compassion differ is in the nature of sentients. Caring is the motivation to *look after*, to prevent harm, and to see the object of ones caring, flourish. So, we can “care” for our gardens, cars, or other or possessions, but if our car was damaged in an accident, we would not have compassion for it because compassion, unlike caring, only applies to suffering. There needs to be a conscious awareness of an experience of suffering. Hence, this brings us again to the issue that it is our knowingness that turns caring into compassion.

#### Using the Functions of the Mind

CFT helps clients in all the four domains, such as working with motives and emotions, developing competencies, and practicing specific behaviors. Using the algorithm engagement and action definition of compassion, CFT offers a brief process map of six ways of supporting engagement and six of taking helpful action. Together, these create courage to engage with suffering, and wisdoms of what is likely to be helpful. Out of this, develops the commitment to work and live “compassionately.” CFT offers psychoeducation into how and why we have tricky brains that are prone to suffering and anti-social behavior; how and why our emotions track the three life tasks; the nature of the autonomic nervous system and the link between breathing practices and other vagal nerve supports, such as biofeedback, diet and exercise; how to ground oneself and one’s body; how to differentiate between different motives and emotions; the value of practicing different visualizations, behaviors and ways of reasoning, and exposure to avoided material aimed at build building courage skills and wisdoms. As clients begin to understand the nature of the minds we all have, these all help to reduce unhelpful blame and shame. They build a self-identity to “live to be helpful not harmful to self and others.”

For engagement, therapists work with the client on understanding what compassion is and is not (and the key roles of courage and wisdom) and developing their motivation to pursue compassion as an approach to mental anguish, and thus to “*care for one’s own and others well being*.” This involves developing a type of mindfulness and *sensitivity*, tuning into suffering and its causes. This then activates a *sympathetic* response. For example, as a client begins to tune into their depression (rather than trying to drug it away), they may begin to feel more in touch with the pain of say loneliness or previous trauma. Their distress would be regarded as a sympathetic response to being tuned into themselves. Clearly, they need to be able to tolerate this otherwise they will just close down again and so *distress tolerance* becomes an important competency. As the client is able to tune in, tolerate, and begin to work with the roots of the pain, they develop an empathic understanding which is a non-blaming and accepting awareness, and open and non-judgmental. These six processes are clearly interdependent and support each other. For example, the more we learn distress tolerance, the more sensitive we can be. Therapists will have a range of evidence-based interventions for helping clients with those competencies common to many therapies, but all the time with the motivation and the orientation for compassion. So, for example, the therapist might have taught soothing rhythm breathing as a grounding in the body process and the stepping into the compassionate self and mind state with a clear compassion intention and focus recalling on strengths and wisdoms, before engaging with deeper sensitivities into (say) trauma. Or they might have explored the three major emotions (see Figure 4), bringing compassion to each emotion before engaging with the origins of a problem. In addition, the client and therapist would have practiced compassion motivational focusing as the context for physiologically grounding the work.

Courageous engagement processes are important, but there are also the wise action processes. Just as when we go to a medical doctor with a broken arm, their understanding of our pain and acceptance of our pain is important, but we hope they also have the *wisdom of knowing what to do*. So, CFT engages in a lot of psychoeducation to help clients develop the wisdom of how and why the brain is tricky and then develop competencies for processes such as mindfulness, how to use our minds for imagining things that are helpful, to learn perspective taking and reasoning in ways that are going to be helpful, to behave in ways that are going to be helpful, to use our bodies to support our minds, such as using breathing exercises or maybe taking physical exercise or attending to diet or learning assertiveness. The feelings we have would be dependent upon the context of the compassion we are doing. As clients become more skilled in the wisdoms of working with their sources of suffering, they are also able to begin to transform suffering. Note that individuals can be good at some compassion processes, but not others. For example, some people may be very empathic, but not so good at compassionate reasoning. Others may be good at some qualities, but they cannot ground themselves in their bodies to give their compassionate mind a chance to function. Others may have beliefs that compassion is weak or you cannot experience rage if you are compassionate.

**Figure 6:**
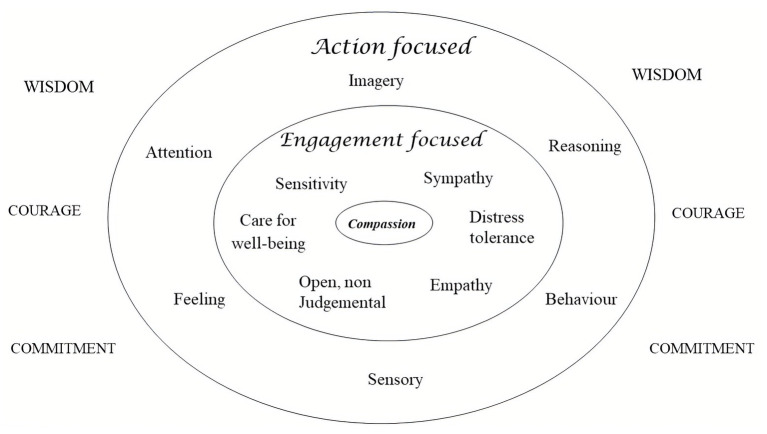
Domains for therapeutic engagement. Adapted from Gilbert (2009a) Compassionate Mind with permission from Little Brown.

Given that these are the core processes for CFT, after a number of disappointing starts, they were developed into a self-report scale (Gilbert et al., 2017). The scale measures engagement and action processes with three orientations: for self, from others, and two others. It has now been used in a number of studies and recently predicted changes in HRV following CFT (Steffen et al., 2020). The six competencies for engagement and six for action are only guides and are given in Figure 6. Together these give rise to commitment, courage and wisdom to acknowledge and address suffering.

#### The Flows of Compassion

Many psychological processes have flow. Consider anger. There is the anger we can feel to other people, the anger we feel from other people to us, and the anger at oneself. Similar for motives of sexuality or cooperation. CFT pays attention to all *three flows* of compassion. There are some conditions where learning compassion for others is important for relationship building and quality. There is also evidence that people who are fearful of *receiving compassion* are vulnerable to mental health problems (Hermanto and Zuroff, 2016; Kirby et al., 2019). The reasons for this are clear, because they cannot use others as a secure base or safe haven and if anything, see the world as a threatening and withholding place. And of course having compassion *for oneself*, rather than being harshly self-critical or self-harming is important. All 12 processes outlined in Figure 6 can be used in each of the flows. For example, when we are sensitive to receiving compassion, we are sensitive to the fact that other people are being sensitive to us and are trying to be empathic, they are able to tolerate our distress and they will work with us to find ways to be helpful. Feeling connected in this way helps us to feel less lonely and even a sense of hope or gratitude; we can move into experiencing more social safeness. When we are being compassionate *to others* then again, we are learning how to be sensitive, empathic, and work out what is helpful. In psychotherapy, sometimes we have to develop the skills for a particular direction. For example, people might be very empathic to others, but not to themselves or they might be able to be very wise for others, but not to themselves and vice versa.

CFT has specific exercises and behavioral practices to address each of these. For example, helping people explore and work with feelings of receiving compassion, they are invited to create an image or concept of an ideal compassionate other relating to them. It is the same principle when people use imagery to stimulate sexual feeling. It is body activation CFT is interested in, rather than clarity of images. So, we guide clients to use compassion images to stimulate care-compassion physiologies, and thereby, opening up certain areas of the frontal cortex. This links very much to the priming attachment type research noted above (e.g. Gillath and Karantzas 2019). The act of imagining and thinking what qualities that image would have is itself a therapeutic process; exploring whether they can trust their own image; practicing generating dialogues with the image; working on misunderstandings about the image such that the image does not want to know about the dark side of the mind, whereas, in fact, it is exactly the opposite – that is partly why we use it; our compassionate images are to help us with our suffering, unruly emotions, and desires. Rather than just *reasoning*, using imagery creates a more natural form of reasoning, which is dialogic. So, we can imagine interacting and communicating with our compassionate image (Lee, 2005; Gilbert, 2020a).

Developing compassion for others can involve empathy training (Luyten et al., 2020) or practicing doing one compassionate act for a person each day or sometimes gratitude focusing. Also, forgiveness can be an important part of developing compassion for others, particularly if they have been harmful to us. Crucially, we recognize that we can forgive people even if we do not like them (Enright and Fitzgibbons, 2015).

#### Compassion and Self-Identity

At the center of the Figure 3 is self-identity. Although a huge subject obviously, self-identity is related to the experienced patterning of our motives and emotions; what we value, what frightens us, what inspires us, and what we aspire to become (Taylor, 1989). In CFT, the identity is compassion; *to live to be helpful not harmful to self and others*, not to be a cause of suffering. This has overlap with the concept of Buddhist pursuit of bodhicitta and also the 8-fold pathway of compassionate living (Gilbert, 2017c).

In the top left and right of Figure 3 is recognition that the social and ecological contexts in which we live impacts on whether we experience the world as harmful or helpful; benign or hostile. The last 5,000 years of human history highlight our enormous capacity for cruelty and harmfulness, in our wars, tortures, painful executions slavery, exploitations, makers of nuclear and biological weapons, that sit alongside the extraordinary developments in medicine, for example, ridding the world of smallpox (Gilbert, 2018). CFT conveys this to clients by indicating that we are gene built, easily injured, short lived beings with a range of potential motives and emotions that we did not choose (e.g., for terrors, panic, rage, depression, lust, and joy). We have brains and bodies built for us, not by us. Nor did we choose the versions we are (Gilbert, 2009a). We suggest to clients that if “I as therapist had been kidnapped as a 3-day old baby into a violent drug gang, this version of Paul Gilbert would not exist.” A very different version with different epigenetic profiles and a physiological patterning of motives, values, and behaviors would be here. The client is invited to hypothesis what I would be like, what I would feel, what would my aspirations and values be, how I might react to someone threatening me; how my body would work etc. The intention is to use oneself to help clients recognize that *we are all* partly socially created versions of ourselves that we never chose. Maybe we could learn how to create inner versions of ourselves *that we can choose* to develop and which will organize our minds in ways that are more conducive to ours and others well being. This is an important de-centering and de-shaming guided discovery. To be kidnapped would not be something I had chosen, any more than any of us choose to be born into the families, social contexts, and cultures we are. The contextual development for a compassionate mind, therefore, is scientific wisdom about the unchosen nature of our biological minds, and as the Buddha (might have) said: that without some kind of mind training and awareness, we are at the mercy of these inherited and socially constructed passions of the mind.

One of the great challenges for compassion training is to incorporate scientific understanding of the mind so that we can begin to break our conditioning. As the science fiction series Westworld so brilliantly indicated, coming to understand the nature of our conditioning is crucial for our development. Part of the point of mindfulness is to help us unstick and decouple ourselves from the experiences of our biological mind as if they are somehow eternal or substantial. We can use mindfulness to start to become aware when different aspects of our programming, such as anger, anxiety or various desires, start to arise. We can be mindful of the fact that we have found ourselves in a male or a female body, living in this ethnic or that ethnic group, but we do not need to over identify with these processes. Crucially, we have greater insight into how the mind is constructed, and hence, why it is so easy for us to be harmful. Evolution cares nothing about harmfulness if it advances gene replication. These insights are actually our challenge as a species.

This leads directly to (see bottom of the Figure 3) the fact that motives, emotions, competencies, and behaviors are all subject to *facilitators and inhibitors* (Gilbert et al., 2011; Gilbert and Mascaro, 2017; Kirby et al., 2019). There are things that will bring out the best and the worst in us (Sapolsky and Share, 2004; Sapolsky, 2017). A main reason for mind training is to have insight into the nature of the mind, so that we do not act out the dark side of the mind.

#### Contextualizing Compassion

Taking a contextual approach also highlights that compassion is not one process but is very dependent upon context. For example, the skills and emotions of a firefighter about to enter a burning house will be very different to say a counselor with a bereaved person, or liberation activists fighting injustice. Although it is sometimes said we need a calm mind to be compassionate, it all depends on the context and what we are developing compassion for. When courageous action is called for, we need a focused mind, not necessarily a calm mind. There is an old story that in training bomb disposal experts would we choose: an anxious or a non-anxious person and the answer is always choose the anxious person (although not out of control anxiety!). Note, too, that some individuals can be extremely compassionate in certain conditions such as risking their lives to save others, but not necessarily that empathic or compassionate with more emotional issues. Our brave firefighter might not be the most empathic partner or parent. And a wonderful loving parent might not make a good firefighter with steady nerves. The role of warmth may be crucial in close relationships, and some forms of psychotherapy, but not in the urgencies of the rescue services. This tells us that compassion is multifaceted, that different people can be skilled in different facets, in different contexts, and should not be seen as unidimensional. At times, compassion can be morally tricky. What is the position of armies who believe they are protecting the population when they go to war?

Self-compassion can also be contextual (Zuroff et al., in press). We may have a lot of compassion if we have lost our jobs through COVID which is not our fault or broken a leg, but not when it comes to making mistakes or getting too anxious to go to the interview for a new job. If we are frustrated or disappointed in ourselves, we can become critical not compassionate. Note too, that although some models of self-compassion combine self-criticism, shame and kindness in its conceptualization and measurement, for clinical purposes CFT keeps these separate. This is partly because there is a large amount of literature on the link between shame and self-criticism and their complexities on mental health problems. In addition, shame and self-criticism operate through the threat system, whereas kindness operates through the soothing and safeness system. If you combine them, it is unclear which process, physiological and psychological, is driving mental health problems. In addition, they may relate to secure base and wellbeing in different ways (Gilbert et al., 2017).

Because so much interest in compassion has grown up around the Buddhist meditative traditions the *courage aspect* and the *activation aspect of compassion*, which can be so important in psychotherapy, is sometimes not emphasized. Also, concepts like compassion can be confused with concepts like love or kindness which are quite different (Gilbert et al., 2019). When we follow an evolutionary approach, we can see that (western concepts of) love, like assertiveness, and empathy are *ways of being compassionate* in certain contexts, but in many ways the strongest compassion is for those that we do not “love.” Kindness is also a signal of friendliness and helpfulness – in other words, it is a safeness *signal* indicating one’s motivation orientation from the other or to the other or oneself. Hence, facilitating kindness in therapy is extremely important because it creates the conditions for experiencing a secure base and safe haven, creates trusting and affiliative relationships, stimulates oxytocin and vagal tone, and increases empathy and thoughtfulness. The main difference is that kindness does not require us to have courage to engage with suffering and, when we do kind acts, these tend to be enjoyable; whereas compassion often involves us having to bear some degree of suffering or unpleasant emotions (Gilbert et al., 2019; Mascaro et al., 2020).

A contextual approach also suggests that compassion is a dimensional process with callousness (Gilbert, 2018). Whereas compassion is sensitivity to suffering and wanting to do something about it, callousness is an insensitivity to suffering with an indifference to it. It differs from cruelty, which has the enjoyment of suffering. There are many dimensions that push us from compassion into callousness and self-interest. Perhaps one of the worst examples is slavery, but there have been many instances, where corporations have shown a callous indifference to the ecology they are exploiting and harming and those who work for them (Bakan, 2012). Callousness and indifference to suffering in any form of relationship is problematic. So, when we have a whole economic system based on promoting self-interest and callousness, it poses serious challenges for compassion and sharing Gilbert (in press). Callousness can be a serious problem in psychotherapy, particularly in the forensic services and in those who have psychopathic and narcissistic personalities (Shirtcliff et al., 2009). Importantly, in childhood parental warmth and caring (especially distress sensitivity and positive regard) can offset callous traits (Wright et al., 2018). While compassion training seems to be helpful for individuals who are reasonably orientated that way [studies are typically on WEIRD (White, Educated, Industrialized, Rich, and Democratic) populations; Henrich, 2020], we have yet to discover what kind of compassion training will help narcissistic individuals and organizations become less callous, self-entitled, and exploitive. In many ways, because these are individuals that often manipulate themselves into power, these are the individuals for whom we need to find ways of engaging them with and supporting compassion training. Nonetheless some inspiring work developing CFT in forensic populations have shown that it may l have potential to reduce psychopathic traits in some people (da Silva et al., 2020).

### Switching Motives

From an evolutionary point of view, motives organize emotions, competencies, and behaviors. There is a general view that compassion is a social process, that was especially adaptive in relatively small, stable early hunter-gatherer groups. The switch from a caring and sharing hunter-gatherer lifestyle into a more vicious competitive tribal one was consequences of agriculture (Gilbert, in press; Ryan, 2019). However, historically there have been times when we have tried to get back to a more caring sharing world. For example, researchers have highlighted the fact that following World War II ambitions to create a fairer, more just society, flourished resulting in the building of National Health Services, better education, and services generally. Taxes were high; however, the gradual eroding of that ambition with desires to pay lower taxes and hold more of one’s personal resources under neoliberalism, western society has increasingly focused on competitive motives in education and life in general (Sachs, 2012; Twenge Miller and Campbell, 2014; Curran and Hill, 2019; Ryan, 2019; Gilbert, 2020b). The movement into a more competitive society is also associated with increasing vulnerabilities to mental health problems (Curran and Hill, 2019; Ryan, 2019). This is partly because competitive motives orientate attention, reasoning, sense of an “individualised self” in ways that tend to increase self-focused attention, social comparison, envy, shame, striving for more and better, and self-criticism for failings (Curran and Hill, 2019; Wetherall et al., 2019). Such increasing competitiveness has been at the expense of other prosocial motives. In 2014, Harvard School of Education reported on a study of 10,000 middle class and high school students from 33 schools.^1^ They found that the majority of children prioritized personal success and achievement over caring for others and that concepts of fairness are not given high priority. Many of the students surveyed admitted that they have been prepared to cheat to get on. Students also suggested that their parents and teachers value personal achievement over caring and sharing. The authors believe this indicates a change over the last 20 years or so toward a more intense, individualistic, and personal competitive focus rather than on community values. This change has occurred with prioritizing academic performance as a main indicator of a school’s worth rather than moral or prosocial development. Consistent with this, studies of change in narcissism over the last 20 years come to similar conclusions (Twenge et al., 2014). They report that these changes have major consequences for lowering empathy, concern for others, civic engagement, and general community prosocial interest. In my own research (Gilbert, in press; Gilbert et al., 2007, 2009a) exploring why people compete, and the phenomenon of “competing to avoid inferiority and rejection,” our studies found those vulnerable to depression and anxiety feel they need to compete to avoid being seen as inferior and rejected. In addition, some forms of competitive behavior, particularly between groups orientate us toward harmful violence and oppression (Perry et al., 2013).

**Figure 7:**
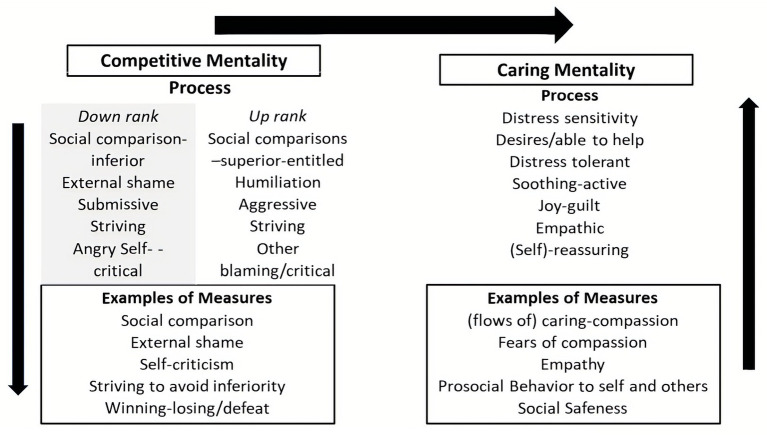
Outline of the competitive vs. caring social mentality as a therapy focus © Paul Gilbert.

As competitive motivation becomes more intense, it can incline to intensify two basic strategies (Gilbert and Basran et al., 2019). A *down or lower rank* strategy that can be seen in many species involves subordinate and “better safe than sorry” threat sensitive lifestyles, careful self-monitoring of social risk, manifested in humans as social comparison, concern with personal inferiority, needing to prove oneself attractive, competent and desirable, with sensitivity to failure, rejection, and exclusion (Gilbert et al., 2007, 2009a). The other strategy is *up-rank narcissistic*, associated with more impulsive and challenging behavior in animals, and in humans, a sense of superiority and entitlement, positive social comparison, blame others for setbacks, and a preparedness to exploit others to benefit self and kin. Interestingly, hyper-competitiveness and “competing to avoid inferiority” are also associated with this group (Basran et al., 2019). Hence, some competitive strategies can indicate concerns with being rendered inferior. The fallout from these competitive motives is very common in mental health difficulties. As indicated in Figure 7, there are various measures that tap into these different aspects of competitive motivation.

As we have seen however compassionate motives, on the other hand, evolved for completely different functions and brought with them completely different sets of physiological systems. Whereas intensifying competitiveness increases threat sensitivity and stress, and divides people, in contrast caring and compassion do the opposite; they partly evolved to regulate threat processing and bring people together (Brown and Brown, 2015, 2017; Mayseless, 2016). Developing compassion for self and others facilitates a different type of physiological organization as well as self-identity and ethical values. This is depicted in Figure 7.

As a general principle then CFT is about *motivational switching*; that is helping people to tune into and recognize the competitive motives/pressures within themselves, for example, the drive to achieve, to avoid failing, the linking achievements with a sense of personal and social worth and identity, worries about being accepted and wanted, the self-criticism that can come out of feeling one is failing (Castilho et al., 2017). As a motive focused therapy, CFT helps clients recognize these two powerful ancient strategies for coping with competitive issues, and how we can get caught in them through no fault of our own. CFT helps people compare and contrast competitive motives with caring motives, and facilitates a number therapeutic stages “for motive switching,” that is gradually shifting our interests attention and focus toward a compassionate orientation to the pressures of living rather than just a competitive “succeed or fail” one (Gilbert, 2000, 2010; Gilbert and Choden, 2013).

## Core Therapy Issues: Fears, Blocks, and Resistances

### Fears, Blocks, and Resistances

As a result of difficult backgrounds, giving and receiving care can be very tricky. This is partly because caring strategies evolved in, and work in, trusting caring environments, less so hostile, unpredictable or neglectful ones. For many of our motives and emotions, we can have fears, blocks, and resistances (FBRs) to them. Fears relate to the anticipated effects of engaging with the motive or emotion; blocks are usually to do with unfamiliarity and confusion or lack of opportunity for experiencing them; and resistances can be seeing the consequences as too costly (Gilbert and Mascaro, 2017). There are now many studies on how fears of compassion (Pauley and MacPherson, 2010; Gilbert et al., 2011) are linked to mental health problems (for a major meta-analysis of this scale see Kirby et al., 2019). In the original studies Gilbert et al. (2011) found it is primarily receiving compassion from others or self rather than giving compassion that are associated with mental health, attachment difficulties and self-criticism. This has been confirmed in a recent meta-analysis (Kirby et al., 2019). Hermanto et al. (2017) showed that the fear of receiving compassion from others was especially linked to mental health difficulties. Matos et al. (2017) and Di Bello et al. (2020) found that fears of compassion, especially fears of receiving compassion from self and others were associated with poorer HRV. Ebert et al. (2018) found that in people with borderline personality difficulties the fears of compassion scale were associated with more difficult childhoods and also lower blood levels of oxytocin. Basran et al. (2019) found that traits such as ruthless ambition and hyper competitiveness were associated with fears of compassion to others. Given these individuals seek positions of power this is obviously a concern (Gilbert and Basran, 2019) especially since Akmal and Foong (2018) found fears of compassion to others was associated with callous traits.

When it comes to compassion *as a therapeutic process* some of the problems can be linked to simple misunderstandings about the nature of compassion and confusing it with “having to be nice, kind or loving or making one weak, or one must not get angry.” Confusions with love are common because of how the internet links western concepts of love with Metta. Indeed, Grammarly software changes compassion into love and kindness. Metta actually means benevolence, open friendliness, and a wish for all sentient beings not to suffer; to wish others well (Ricard, 2015). As most dictionaries will attest love in the west means *liking and wanting to be* with and this will be the concept in the client’s mind. However, benevolence and wishing others to be free of suffering does not require that we love them; a firefighter rescuing a family does not need to love or even like the family. These concepts can be a block in therapy. And when we learn forgiveness, we do not have to love the people we have forgiven; that is a fundamental misunderstanding (Enright and Fitzgibbons, 2015). Self-love, in contrast to self-acceptance or self-insight is usually associated with narcissistic preoccupation. The reality is that we have very messy, difficult minds and it is our courage and wisdom and dedication to live helpfully, not harmfully that is crucial.

Clarity over definition and using guided examples (e.g., “how would we help a friend who was suffering from X”) can help here, because clients often note that they would be sensitive to their friends suffering and then they would try to work out how to be helpful. Once they have explored that example, then that is the basis for sharing the definition given above with them and distinguishing it from kindness, love and so on. In addition, as noted above, people can block out on compassion if they do not like or trust the person who is seeking compassionate help or who would be offering compassionate help. Similarly, for themselves, clients can find it difficult to be compassionate to the things they are ashamed of, their own dark sides or things they hate about themselves. Pauley and McPherson (2010) found that depressed people could see the value of developing compassion and would like to, but felt unable to, partly because of a lack of deserve and high levels of self-criticism and dislike (see also Castilho et al., 2017). Lawrence and Lee (2014) found similar and in a qualitative study found FBRs to compassion were linked to the fear of giving up self-criticism, loss of an identity, and that compassion felt “alien,” and untrustworthy and one to be feared. They also outlined the unfolding steps on their clients abilities to understand, practice and integrate compassion into their way of living.

Other very common sources of FBRs are linked to early life history and the serious ruptures in the internalization and maturation of care focused processing systems and lack of experiencing a safe haven and secure base. Matos et al. (2017) found that fears of compassion, particularly from others and to self were associated with memories of high shame and low parental warmth. Paranoid thoughts were more associated with fears of compassion to others. Various forms of abuse (Lippard and Nemeroff, 2020) and other common histories for people with mental health problems (Van der Kolk, 2015) indicate that either they did not experience feeling a sense of security or safeness and “loving” empathic engagement with developmental needs being met, and/or as they move through life they continued to feel socially disconnected, “searching for a secure base” (Holmes, 2001; Music, 2019). A number of therapies highlight the fact that experiences of shame and lacking a sense of “being held positively in the mind of others” is a common source of feeling separate and disconnected. Indeed, central to CFT is to help generate a sense of connectedness and interdependence as well as self-regulation partly by working with shame and shame trauma. The fear of shame and being “exposed” means that we cannot turn to others for “secure base or safe haven” functions (Gilbert, 1989/2016, 2007a,b; Holmes and Slade, 2017; Music, 2019; Schore, 2019). Facilitating compassion to specifically address shame can be an antidote for the sense of alienation from self and others (Gilbert, 2000; Gilbert and Irons, 2005).

### Classically Conditioned Fears

**Figure 8:**
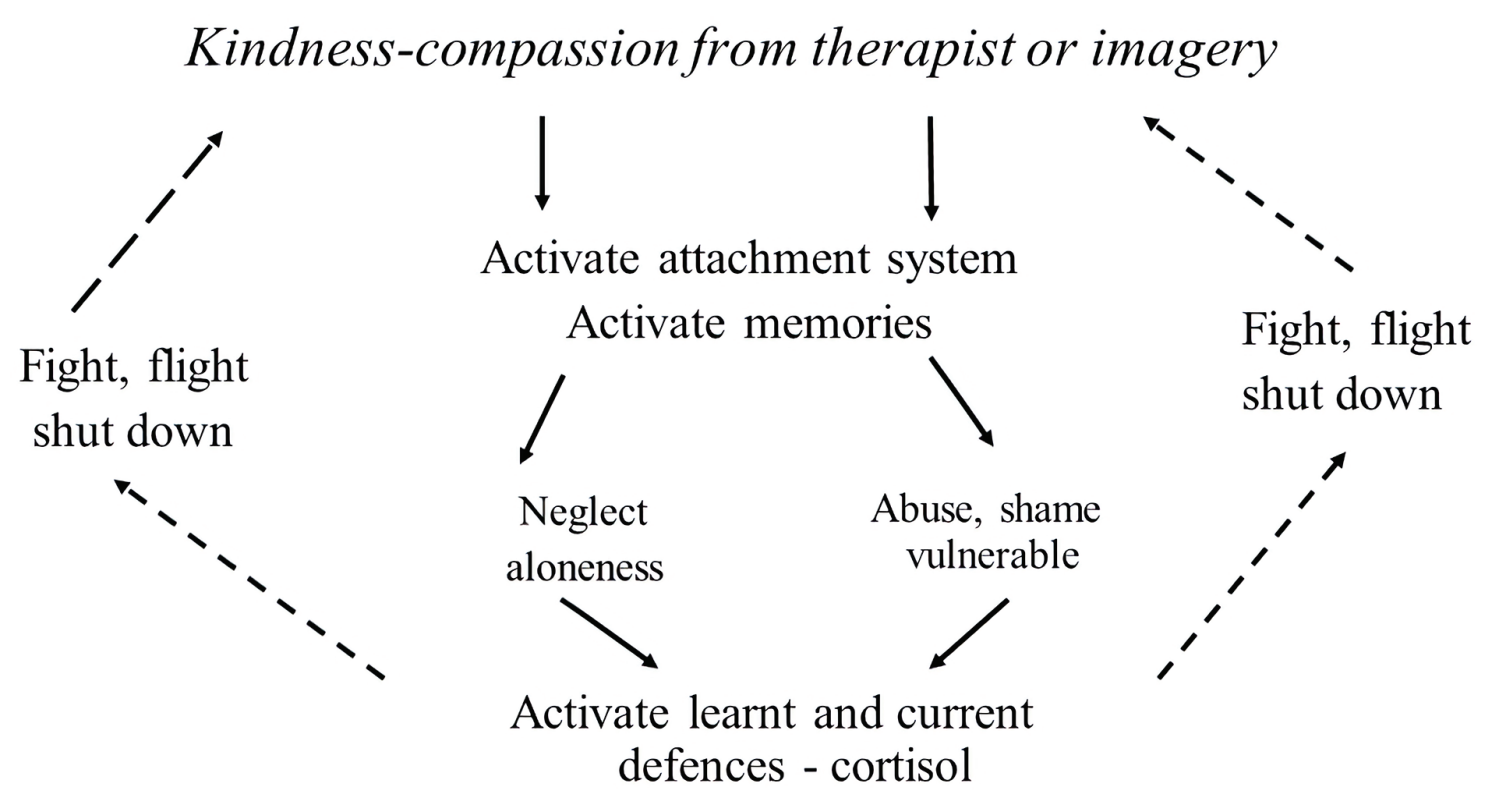
Fears of compassion and attachment history. Adapted from Gilbert (2009b).

Many years ago, I attended a lecture at the Tavistock Clinic in London reflecting on Bowlby’s legacy. Delegates watched a recorded interview made some years earlier where he spoke about how, sometimes when he was empathic and understanding to his clients, trying to help them feel cared about, they would become anxious or angry. This was certainly my experience too. He suggested that if people have a compromised attachment system then they tend to respond to a “care signal” through that threat sensitive lens. One way of thinking about it is in terms of *classical conditioning and emotional learning*. For example, imagine you enjoy holidays, looking forward to them you get excited. Then one year, you are badly beaten up and robbed. What happens the next year when you see advertisements for holidays? Chances are you do not remember all the wonderful holidays you have had, but the trauma will come back. Imagine having somebody take a sexual interest. If your sexual experiences have been primarily positive, this may be enjoyable, but if your experiences have been traumatic, that could be very frightening to you. Hence, stimuli of caring and compassion will open the “attachment systems,” and emotion memories of being cared for. If neglect and trauma are in that social mentality memory system, then that is what will start to come back. In addition, the “seeking aspect” of the motive system may generate rage or grief that can be fearful or overwhelm clients. So, it is easy to unintentionally trigger trauma, neglect and loneliness memories through compassion training. One of the simplest ways of dealing with that is simply to share it with our clients so they can make sense about what is happening for them, normalize, and validate “It makes perfect sense why compassion is scary for you” and then begin to work on ways to collaborate on how to detoxify the care seeking system and desensitization trauma. Figure 8 depicts this process.

How we address these issues with clients is important, and caution is needed. For example, comparing resistance to “a fire backdraft” as has been suggested by some can create frightening imagery for clients. So CFT tries to help the client recognize that what is feared is also the source of their healing. So, CFT invites a shared drawing of figures like Figure 8 with clients, building them together, and discussing reflecting and exploring as you go. Time is given to making sense of why compassion feels dangerous or pointless. In addition, the therapist labels fear and resistance as *an intuitive wisdom*; that we sometimes need small steps. We do not learn to swim by jumping off the high diving board. So, we can re-label fears and resistances as *cautions and protections*, but we need to know what is being protected. Hence, at each stage, we can reflect with the client the potential pain and fear of compassion, validating along the way, but also indicating that it is possible to work with compassion, noting the advantage of doing so, for example, having access to a birth right for emotion regulation through the vagus nerve and building on one own inner courage.

In addition, helping clients with the abilities to *feel compassion* can take time and can be linked to the abilities to tolerate sadness and to grieve (or rage) for the love they did not have, but wanted (Gilbert and Irons, 2005). Over the years, I have worked with many who had lived with a long sense of disconnection and loneliness, struggling to achieve things, or to do things to make them feel worthy of love and being wanted. For some clients, there is intense grief; for others, there is rage; and sometimes, it is useful to invite clients to look at the big three threat-based emotions, of anger anxiety and sadness to be able to differentiate them and process them as linked to their FBRs (see Figure 4). Hence, when individuals really struggle with compassion, the therapist may recognize the client is not in a position to begin to move down that road and first has to do preparatory work in the care and attachment system. This may involve grief work the balance of teaching how to be “safely embodied” with engagement distress and suffering is not always easy (Rothschild, 2000; Van der Kolk, 2015). Because FBRs are very common in CFT, and at times are frightening to clients, it is important that the therapist recognizes and develops ways to validate this to see these as protections rather than problems, but at the same time not engage in avoidance of compassion cultivation. This because if clients cannot develop the care and compassion motives and competencies, then they are cut off from all of those potentially helpful emotion regulating processes linked to secure base, safe haven, and their physiological substrates.

## Bringing Things Together

**Figure 9:**
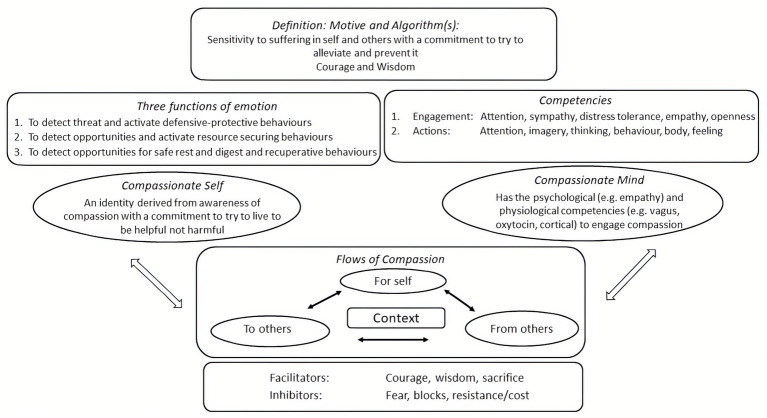
Overview of core processes in CFT © Paul Gilbert.

Figure 9 gives a quick overview of a set of processes involved in CFT.

First, we can identify the basic (evolved) algorithm in terms of the stimulus sensitivity (which is suffering, distress or need), and the responses (which are for alleviation and prevention). Throughout, this paper has highlighted that engaging with mental suffering and distress will require building courage (Bratt et al., 2020), Hence, the cultivation of *courage* to help work with suffering, be it past traumas or current anxieties and depressions, is essential (Rachman, 1990). In addition, understanding how the psychophysiological mechanisms of compassion, particularly as they support secure base and safe haven, give rise to internal states of courage is key and discussed with the client (Gilbert and Choden, 2013, Porges, 2017; Bratt et al., 2020). Because we have complex human cognitive competencies, we can develop wisdoms for compassion. Wanting to be a doctor is a good intention but is useless if we do not study and learn skilled ways of healing. CFT shares with clients why our evolved and socially shaped minds are tricky to navigate, prone to mental distress, and harmful behavior. We help clients to de-shame and de-centre from the processes of a biological mind but learn how to take responsibility for its outputs and behaviors. In both the contemplative traditions and CFT, part of the practices are to help individuals gain insight into the nature of the mind, to become “mind aware and mind wise.” Consequently, the first stages are recognizing the basic algorithm for compassion and its roots in the process of developing the client’s courage and compassion.

At the next level, we help the client begin to explore the textures and climates of their emotional lives, noting particularly the emotions that are difficult, avoided, or over engaged. CFT helps clients think about the three *main functions of emotion*: threat protection defense, resource and reward seeking, and resting gettling, safeness and contentment. We can also explore with clients the link between the autonomic nervous system and other physiological systems and their abilities to emotionally regulate. The right-hand side of Figure 9 shows the 12 processes depicted in Figure 6.

As clients begin to understand the nature of mind, the ease by which we can experience mental suffering, the nature of the tricky mind, and how compassion can reorganize our minds, compassion becomes a focus for the identity of the self (Gilbert and Choden, 2013; Gilbert and Basran, 2018). The client begins to adopt the idea *to live to be helpful, not harmful* to self, and others and becomes more sensitive to the ease by which we can inadvertently be harmful. In addition, the joyfulness of helping and compassion is highlighted.

To live to an identity, however, requires us to have the mind and body to do it. We may be very keen to climb a mountain or run a marathon, but we need the body to do it. CFT argues that this is similar in psychotherapy. People can want to be courageous or to face their difficulties and live compassionately, but they just do not have the physiological resources to do it (Cozolino, 2017; Porges, 2017; Schore, 2019). The threat system is very sensitive. Hence, the importance of all those practices that help to build an inner secure base and safe haven supported with their physiological development. Throughout the therapy, we constantly anticipate and address facilitators and inhibitors of all these processes.

## Conclusion and Summary

This paper has highlighted the evolution of caring and its transition into compassion. In addition, it has explored the psychological functions of caring and compassion motives that are salient to psychotherapy for helping people in distressed states in mind. We have addressed the fact that compassion is not one process, but a multi-faceted process, where people can be good at some aspects of compassion or some competencies of compassion, but not others. We noted too that caring compassion evolved to be focused on kin or reciprocating relationships, but then we can extend it through developing a compassionate identity and by training the mind in the insights and competencies of compassion which opens up new opportunities for that identity. Given that the human condition is one of suffering from injury, disease, getting old, decaying, and dying, and experiencing many setbacks and losses along the way, the therapeutic focus is how to deal with the sufferings we experience in life. Along with addressing suffering, however, CFT like many other therapies also aims to help people develop their own sense of meaning, happiness and to flourish. In our relationship with our clients, we help them see that “we see them” as fellow human beings on this tricky journey called life, with different versions of ourselves. So, the therapist is not there to “fix things gone wrong” but rather to help people re-pattern rewire and reprogram themselves, in ways that are more conducive to dealing with suffering and promoting well-being.

With the increasing awareness of the importance of linking psychological processes to physiological states and social relationships many new avenues of therapies are emerging that include: direct vagal stimulation and balancing the ANS with diet and exercise, using sounds, chanting, breath training, yoga and dance, various forms of biofeedback and also the use of psychedelic drugs that can promote experiences of deep social connectedness. In addition, more focus is going to be on developing compassionate community living. Research is revealing new ways to promote insights into the nature of mind, promote mental well-being and prosocial behavior. How these might help those with more narcissistic and callous traits become more compassionate is an exciting research question. Hence, the psychotherapy of the future will look somewhat different to what we have today and be a much more biopsychosocial process (Mascaro et al., 2020) and concerned with ethics and pro social behavior in all dimensions of social relating. For now though some of the conditions for CFT include:

1. A central theme of CFT is clear definitions and understanding of compassion. This is because there are many different concepts of compassion on the internet and clients come with all kinds of ideas that are not conducive to compassion as a therapy. Hence, the focus on compassion is as an algorithm that at its heart is based on wisdom and courage. Moreover, compassion is not one thing, so we might need to develop different types of competencies, abilities, insights, and wisdoms, for different types of problem. This is often new to clients.
2. During the course of the therapy, we cover a functional analysis of the various safety behaviors clients are using, including different forms and functions of self-criticism. We can contrast that with the functions of caring behavior and its derivative compassion and how to turn to compassionate self-correction. The evolved functions of caring on *psychology* are depicted in approaches such as attachment theory and other developmental models. Caring evolved to provide a *source of protection, a secure base* to offer support, and encouragement and guidance for learning and flourishing, and *safe haven* that offers ways of regulating threat (and sometimes drive) emotions that have become destabilizing or overwhelming. The compassionate imagery reasoning and behavioral exercises, and also compassionate self are cultivated with and for these functions.
3. CFT uses evolution insights to help clients depersonalize and decenter from their inner experience. We are a species among many millions, created by genes to pursue certain life tasks, navigating the myriad of potentially strongly felt emotions, mood shifts and desires that are built into the brain and can be extremely difficult. Therapists also use the idea of what they (therapist) would be like if they had been kidnapped as “three day old babies” into violent gangs. We help clients recognize they have a certain type of consciousness, which in itself is empty of content; the source of content comes from our gene built and socially textured brains and phenotypes; these are mostly a game of chance and luck. We are all just versions of biological beings who have been sculpted by the environment. There is no real self here at all, just patterns of electrochemical activity patterning experiences in consciousness. These insights have important de-shaming effects facilitating a greater preparedness to look at some of the dark or frightening sides of the self without blaming oneself for them.
4. Hence, CFT helps clients develop a sense of common humanity and an appreciation that *we all* just find ourselves here, with a body and mental contents and flow (needs, passions, and fears) that *have been built for us, not by us*. Our life tasks are to try to work out “our programming” so that we can often override it and be helpful, not harmful. Empathy training helps in the recognition that we can unintentionally end up in states of personal suffering and also cause it to others.
5. Once we acknowledge we have this tricky brain, CFT helps clients recognize that if we become *mindful and mind aware*, we become more sensitive to the different textures of the mind. This enables us to become more aware of what motives and emotions are running in our mind and whether we want to let them. Mindfulness in CFT, therefore, is to develop mind awareness into a *biologically and social textured created minds*. Then, we can recognize when to switch attention, thinking, emotions, and motives away from the unhelpful to the helpful.
6. CFT helps clients recognize the importance of insight into our multifarious “multiple selves” and often *conflicting* nature of the mind, and hence, the need to develop mind awareness and *abilities to differentiate the complex of motives and emotions and beliefs*, the texture the mind. Mind awareness gives rise to compassionate wisdom and the issue of integration.
7. Mind awareness goes with body awareness and also body cultivation. CFT offers insight and guidance into how to train/use the body to support the mind. For example, the importance of developing vagal tone, how to use breathing exercises to settle and ground the body and mind, how to use posture and exercise, pay attention to diet which can influence the vagal nerve, learning to process threatening information in ways contextualized with a secure base and safe haven, and how to increase certain activating positive emotions and helpful desires.
8. CFT offers clients a range of mind and body training practices that include breathing, posture visualizations, meditations, behavioral practices, and other traditional western therapeutic skills such as: compassionate writing and journaling, compassionate acting, using chairs to help differentiation of feelings and motives, compassionate behavioral planning; use of art, music and dance.
9. CFT helps clients to reflect on what is *meaningful* to them, the self-identities they want to foster and carry through life, and how they might like to look back on their life as it draws to an end. Compassion is typically experienced as a source of meaningful action.
10. With clients who had complex developmental histories, perhaps, with trauma or neglect, a lot of the work is around fears, blocks, and resistances. That includes working with problems of distrust, and facilitating engagement with unprocessed emotions. A very common process is *grieving for the life one did not have* and the yearning to feel loved, validated cared for, approved of or admired especially but not only by a parental figure. These are basic human needs and when they are not met, they do not simply shrivel up and disappear, they lurk in us in all kinds of ways.
11. Crucial is to recognize that fears, blocks, and resistances are not a problem; *they are the work*. Sometimes when therapists run into them, they take that as a reason not to do CFT! But like behavior therapists, the more avoidance, the more you help people engage and get behind the fears and resistances. CFT treats fears as ‘intuitive wisdoms of protection’ coded in the body ‐ one just needs to find out, ‘protecting from what’?
12. Finally, wherever possible Socratic inquiries and guided discovery are the way to help people understand the nature of the tricky minds we have got, compassion, and enter into the training exercises. Although there are many psychoeducation and behavioral practices, CFT, like other therapies values the therapeutic relationship; the sharing of life stories and narratives with silences for reflection, gentle nudging towards working with difficult emotions, sharing in the joys of movement forward and the sorrows of setbacks. Clients are monitoring the degree to which the therapist is not just empathic but is motivated to help them and are partners on the journey.

The lead up to CFT has emerged slowly over the past 40 years or so. Some of its origins are in early work on motivational processes, distinctions between threat, safety, and safeness systems and integration with attachment theory (Gilbert, 1984, 1989/2016, 1992a,b, 1993). With the unfolding of years, new concepts of compassion, including Buddhist meditation practices and research on how compassion changes a range of physiological and neurophysiological systems, have all added to our understanding of how care-based motive can operate as major regulating systems of emotions and prosocial behavior (Weng et al., 2013; Singer and Engert, 2019). Although CFT utilizes many cognitive behavioral and other techniques, it has always been rooted in evolution informed behavioral models (Gilbert, 1984, 1989/2016). Thus it is not a third wave of CBT. And the future of psychotherapy is in the biopsychosocial sciences, rather than new versions of particular approaches (Cozolino, 2017; Gilbert, 2019; Schore, 2019; Siegel, 2019). CFT has always been a process therapy and has tried to articulate specific processes of intervention carefully and clearly to put them up for scientific validation. Our outcomes will improve, the better we understand process, and the more we understand what our minds evolved to do and need, the more we will understand process. Finally, we should note that psychotherapy is also about moral and ethical behavior; coming to therapy should also make us more compassionate human beings.

## Author Contributions

The author confirms being the sole contributor of this work and has approved it for publication.

### Conflict of Interest

The author declares that the research was conducted in the absence of any commercial or financial relationships that could be construed as a potential conflict of interest.

## Footnotes

^1^https://mcc.gse.harvard.edu/reports/children-mean-raise

## References

[ref200] Akmal K. M. Foong A. L. S. (2018). Attachment styles: fear of compassion and callous-unemotional traits among juvenile delinquents. J. Child Adolesc. Behav. 6:371. 10.4172/2375-4494.1000371

[ref1] Amodio P. (2019). Octopus intelligence: the importance of being agnostic. Anim. Sentience 4:20.

[ref2] Armstrong B. F. Nitschke J. P. Bilash U. Zuroff D. C. (2020). An affect in its own right: investigating the relationship of social safeness with positive and negative affect. Pers. Individ. Differ. 168:109670. 10.1016/j.paid.2019.109670

[ref3] Ashbach C. Fraley K. Koehler P. Pollton J. (2020). Suffering and sacrifice in the clinical encounter. Bicester, UK: Phoenix Publishing.

[ref201] Bakan J. (2012). The corporation: The pathological pursuit of profit and power. UK: Hachette.

[ref4] Baron-Cohen S. (2011). Zero degrees of empathy: A new theory of human cruelty. UK: Penguin.

[ref5] Barrett L. F. (2017). How emotions are made: The secret life of the brain. New York: Houghton Mifflin Harcourt.

[ref6] Basran J. Pires C. Matos M. McEwan K. Gilbert P. (2019). Styles of leadership, fears of compassion, and competing to avoid inferiority. Front. Psychol. 9:2460. 10.3389/fpsyg.2018.02460, PMID: 30723443 PMC6349715

[ref7] Baumeister R. F. Bratslavsky E. Finkenauer C. Vohs K. D. (2001). Bad is stronger than good. Rev. Gen. Psychol. 5, 323–370. 10.1037/1089-2680.5.4.323

[ref8] Bell T. Montague J. Elander J. Gilbert P. (2020a). “A definite feel-it moment”: embodiment, externalisation and emotion during chair-work in compassion-focused therapy. Couns. Psychother. Res. 20, 143–153. 10.1002/capr.12248

[ref9] Bell T. Montague J. Elander J. Gilbert P. (2020b). “Suddenly we are King Solomon”: multiplicity, transformation and integration in compassion focused therapy chairwork. J. Psychother. Integr. 1–20. 10.1037/int0000240

[ref11] Bloom P. (2017a). Against empathy: The case for rational compassion. UK: Random House.

[ref12] Bloom P. (2017b). Empathy and its discontents. Trends Cogn. Sci. 21, 24–31. 10.1016/j.tics.2016.11.004, PMID: 27916513

[ref202] Boehm C. (1999). Hierarchy in the forest: The evolution of egalitarian behavior. Harvard: Harvard University Press.

[ref13] Bohlmeijer E. Prenger R. Taal E. Cuijpers P. (2010). The effects of mindfulness-based stress reduction therapy on mental health of adults with a chronic medical disease: a meta-analysis. J. Psychosom. Res. 68, 539–544. 10.1016/j.jpsychores.2009.10.005, PMID: 20488270

[ref14] Bowlby J. (1969). Attachment and loss. Vol. 1 London: Random House.

[ref15] Bowlby J. (1973). Attachment and loss: Separation, anxiety and anger. Vol. 2 London: The Hogarth Press and the Institute of Psycho-analysis, 1–429.

[ref16] Bowlby J. (1980). Attachment and loss. Vol. 3 London: Hogarth Press and The Institute of Psycho-Analysis.

[ref17] Brasini M. Tanzilli A. Pistella J. Gentile D. Di Marco I. Mancini F. (2020). The social mentalities scale: a new measure for assessing the interpersonal motivations underlying social relationships. Pers. Individ. Differ. 167:110236. 10.1016/j.paid.2020.110236

[ref18] Bratt A. Gralberg I. M. Svensson I. Rusner M. (2020). Gaining the courage to see and accept oneself: group-based compassion-focussed therapy as experienced by adolescent girls. Clin. Child Psychol. Psychiatry 25, 909–921. 10.1177/1359104520931583, PMID: 32508169 PMC7528542

[ref19] Brown S. L. Brown R. M. (2015). Connecting prosocial behavior to improved physical health: contributions from the neurobiology of parenting. Neurosci. Biobehav. Rev. 55, 1–17. 10.1016/j.neubiorev.2015.04.004, PMID: 25907371

[ref20] Brown S. L. Brown R. M. (2017). “Compassionate neurobiology and health” in The Oxford handbook of compassion science. eds. Seppälä E. M. Simon-Thomas E. Brown S. L. Worline M. C. Cameron L. Doty J. R. (New York: Oxford University Press), 159–172.

[ref21] Buss D. M. (2015). Evolutionary psychology: The new science of the mind. 5th Edn. UK: London Psychology Press.

[ref22] Byrne R. (1995). The thinking ape: Evolutionary origins of intelligence. UK: Oxford University Press.

[ref23] Carter S. Bartal I. B. Porges E. (2017). “The roots of compassion: an evolutionary and neurobiological perspective” in The Oxford handbook of compassion science. eds. Seppälä E. M. Simon-Thomas E. Brown S. L. Worline M. C. (New York: Oxford), 178–188.

[ref24] Carter C. S. Harris J. Porges S. W. (2009). “Neural and evolutionary perspectives on empathy” in The social neuroscience of empathy. eds. Decety J. E. Ickes W. E. (Massachusetts: MIT Press), 169–182.

[ref25] Carver C. S. (2004). Negative affects deriving from the behavioral approach system. Emotion 4, 3–22. 10.1037/1528-3542.4.1.3, PMID: 15053723

[ref26] Cassidy J. Shaver P. R. (2016). Handbook of attachment: Theory, research and clinical applications. 3rd Edn. New York: Guilford.

[ref27] Castilho P. Pinto-Gouveia J. Duarte J. (2017). Two forms of self-criticism mediate differently the shame-psychopathological symptoms link. Psychol. Psychother. 90, 44–54. 10.1111/papt.12094, PMID: 27249062

[ref28] Chance M. (1988). Social fabrics of the mind. London: Lawrence Erlbaum.

[ref29] Colqhoun L. Workman L. Fowler J. (2020). “The problem of altruism and future directions” in Cambridge handbook of evolutionary perspectives on human behavior. eds. Workman L. Reader W. Barkow J. H. (Cambridge: Cambridge University Press), 125–138.

[ref30] Condon P. Makransky J. (2020). Sustainable compassion training: integrating meditation theory with psychological science. Front. Psychol. 11:2249. 10.3389/fpsyg.2020.02249, PMID: 33041897 PMC7518715

[ref31] Cordaro D. T. Brackett M. Glass L. Anderson C. L. (2016). Contentment: perceived completeness across cultures and traditions. Rev. Gen. Psychol. 20, 221–235. 10.1037/gpr0000082

[ref32] Cowan C. S. M. Callaghan B. L. Kan J. M. Richardson R. (2016). The lasting impact of early-life adversity on individuals and their descendants: potential mechanisms and hope for intervention. Genes Brain Behav. 15, 155–168. 10.1111/gbb.12263, PMID: 26482536

[ref33] Cozolino L. (2014). The neuroscience of human relationships: Attachment and the developing social brain, Norton series on interpersonal neurobiology 2nd Edn. New York: WW Norton & Company.

[ref34] Cozolino L. (2017). The neuroscience of psychotherapy: Healing the social brain, Norton series on interpersonal neurobiology 2nd Edn. New York: WW Norton & Company.

[ref35] Craig C. Hiskey S. Spector A. (2020). Compassion focused therapy: a systematic review of its effectiveness and acceptability in clinical populations. Expert. Rev. Neurother. 20, 385–400. 10.1080/14737175.2020.1746184, PMID: 32196399

[ref36] Curran T. Hill A. P. (2019). Perfectionism is increasing over time: a meta-analysis of birth cohort differences from 1989 to 2016. Psychol. Bull. 145, 410–429. 10.1037/bul0000138, PMID: 29283599

[ref203] da Silva D. R. Rijo D. Salekin R. T. Paulo M. Miguel R. Gilbert P. (2020). Clinical change in psychopathic traits after the PSYCHOPATHY.COMP program: preliminary findings of a controlled trial with male detained youth. J. Exp. Criminol. 1–25. 10.1007/s11292-020-09418-x32837458

[ref37] Decety J. E. Ickes W. E. (2009). The social neuroscience of empathy. Massachusetts: MIT Press.

[ref38] Del Giudice M. (2016). The life history model of psychopathology explains the structure of psychiatric disorders and the emergence of the p factor: a simulation study. Clin. Psychol. Sci. 4, 299–311. 10.1177/2167702615583628

[ref39] Depue R. A. Morrone-Strupinsky J. V. (2005). A neurobehavioral model of affiliative bonding: implications for conceptualizing a human trait of affiliation. Behav. Brain Sci. 28, 313–395. 10.1017/S0140525X05000063, PMID: 16209725

[ref204] Di Bello M. Carnevali L. Petrocchi N. Thayer J. F. Gilbert P. Ottaviani C. (2020). The compassionate vagus: a meta-analysis on the connection between compassion and heart rate variability. Neurosci. Biobehav. Rev. 116, 21–30. 10.1016/j.neubiorev.2020.06.016, PMID: 32554001

[ref40] Dunbar R. I. (2009). The social brain hypothesis and its implications for social evolution. Ann. Hum. Biol. 36, 562–572. 10.1080/03014460902960289, PMID: 19575315

[ref41] Dunbar R. (2014). Human evolution: A pelican introduction. UK: Penguin.

[ref205] Ebert A. Edel M.-A. Gilbert P. Brüne M. (2018). Endogenous oxytocin is associated with the experience of compassion and recalled upbringing in borderline personality disorder. Depress. Anxiety 35, 50–57. 10.1002/da.22683, PMID: 28881460

[ref42] Eisenberg N. VanSchyndel S. K. Hofer C. (2015). The association of maternal socialization in childhood and adolescence with adult offsprings’ sympathy/caring. Dev. Psychol. 51, 7–16. 10.1037/a0038137, PMID: 25383690

[ref43] Enright R. D. Fitzgibbons R. P. (2015). Forgiveness therapy: An empirical guide for resolving anger and restoring hope. New York: American Psychological Association.

[ref44] Eyal T. Steffel M. Epley N. (2018). Perspective mistaking: accurately understanding the mind of another requires getting perspective, not taking perspective. J. Pers. Soc. Psychol. 114, 547–571. 10.1037/pspa0000115, PMID: 29620401

[ref45] Fiskum C. (2019). Psychotherapy beyond all the words: dyadic expansion, vagal regulation, and biofeedback in psychotherapy. J. Psychother. Integr. 29, 412–425. 10.1037/int0000174

[ref46] Fonagy P. Adshead G. (2012). How mentalisation changes the mind. Adv. Psychiatr. Treat. 18, 353–362. 10.1192/apt.bp.108.005876

[ref47] Fonagy P. Campbell C. Bateman A. (2017). Mentalizing, attachment, and epistemic trust in group therapy. Int. J. Group Psychother. 67, 176–201. 10.1080/00207284.2016.1263156 38449238

[ref48] Fox J. Cattani K. Burlingame G. M. (2020). Compassion focused therapy in a university counselling and psychological services center: a feasibility trial of a new standardized group manual. Psychother. Res. 1–13. 10.1080/10503307.2020.1783708, PMID: [Epub ahead of print] 32584204

[ref49] Frank E. T. Wehrhahn M. Linsenmair K. E. (2018). Wound treatment and selective help in a termite-hunting ant. Proc. Biol. Sci. 285:20172457. 10.1098/rspb.2017.2457, PMID: 29445019 PMC5829198

[ref50] Fredrickson B. Mancuso R. Branigan C. Tugade M. (2000). The undoing effects of positive emotion. Motiv. Emot. 24, 237–258. 10.1023/a:1010796329158, PMID: 21731120 PMC3128334

[ref51] Geary D. C. (2000). Evolution and proximate expression of human parental investment. Psychol. Bull. 126, 55–77. 10.1037/0033-2909.126.1.55, PMID: 10668350

[ref52] Germer C. K. Siegel R. D. (2012). Wisdom and compassion in psychotherapy: Deepening mindfulness in clinical practice. New York: Guilford Press.

[ref53] Gilbert P. (1984). Depression: From psychology to brain state. London: Lawrence Erlbaum Associates.

[ref54] Gilbert P. (1989/2016). Human nature and suffering. London: Routledge.

[ref206] Gilbert P. (1992a). Depression: The evolution of powerlessness. London: Lawrence Erlbaum Associates.

[ref207] Gilbert P. (1992b). Defence, safe(ty) and biosocial goals in relation to the agonic and hedonic social modes. World Futures 35, 31–70. 10.1080/02604027.1992.9972316

[ref55] Gilbert P. (1993). Defence and safety: their function in social behaviour and psychopathology. Br. J. Clin. Psychol. 32, 131–153. 10.1111/j.2044-8260.1993.tb01039.x, PMID: 8318932

[ref56] Gilbert P. (1995). Biopsychosocial approaches and evolutionary theory as aids to integration in clinical psychology and psychotherapy. Clin. Psychol. Psychother. 2, 135–156. 10.1002/cpp.5640020302

[ref57] Gilbert P. (2000). “Social mentalities: internal ‘social’ conflicts and the role of inner warmth and compassion in cognitive therapy” in Genes on the couch: Explorations in evolutionary psychotherapy. eds. Gilbert P. Bailey K. G. (Hove: Psychology Press), 118–150.

[ref58] Gilbert P. (2005a). “Compassion and cruelty. A biopsychosocial approach” in Compassion: Conceptualizations, research and use in psychotherapy. ed. Gilbert P. (London: Routledge), 11–74.

[ref59] Gilbert P. (2005b). “Social mentalities: a biopsychosocial and evolutionary reflection on social relationships” in Interpersonal cognition. ed. Baldwin M. (New York: Guilford), 299–333.

[ref60] Gilbert P. (2007a). Psychotherapy and counselling for depression. 3rd Edn. London: Sage.

[ref61] Gilbert P. (2007b). “Evolved minds and compassion in the therapeutic relationship” in The therapeutic relationship in the cognitive behavioural psychotherapies. eds. Gilbert P. Leahy R. (London: Routledge), 106–142.

[ref62] Gilbert P. (2009a). The compassionate mind: A new approach to the challenge of life. London: Constable & Robinson.

[ref63] Gilbert P. (2009b). “Developing a compassion focused approach in cognitive behavioural therapy” in Cognitive behaviour therapy, a guide to the practicing clinician. Vol. 2. ed. Simos G. (London: Routledge).

[ref64] Gilbert P. (2010). Compassion focused therapy: The CBT distinctive features series. London: Routledge.

[ref65] Gilbert P. (2014). The origins and nature of compassion focused therapy. Br. J. Clin. Psychol. 53, 6–41. 10.1111/bjc.12043, PMID: 24588760

[ref66] Gilbert P. (ed.) (2017a). Compassion: Concepts, research and applications London: Routledge.

[ref67] Gilbert P. (ed.) (2017b). “Compassion: definitions and controversies” in Compassion: Concepts, research and applications. (London: Routledge), 3–15.

[ref68] Gilbert P. (ed.) (2017c). “Compassion as a social mentality: an evolutionary approach” in Compassion: Concepts, research and applications (London: Routledge), 31–68.

[ref69] Gilbert P. (2018). Living like crazy. York: Annwyn House.

[ref70] Gilbert P. (2019). Psychotherapy for the 21st century: an integrative, evolutionary, contextual, biopsychosocial approach. Psychol. Psychother. 92, 164–189. 10.1111/papt.12226, PMID: 30932302 PMC6593829

[ref71] Gilbert P. (2020a). “The evolution of prosocial behavior: from caring to compassion” in Cambridge handbook of evolutionary perspectives on human behavior. eds. Workman L. Reader W. Barkow J. H. (Cambridge: Cambridge University Press), 419–435.

[ref72] Gilbert P. (2020b). “Evolutionary functional analysis: the study of social mentalities, social rank and caring-compassion” in Making an impact on mental health. eds. Kirby J. N. Gilbert P. (London: Routledge), 4–42.

[ref208] Gilbert P. (in press). Creating a compassionate world: addressing the conflicts between sharing and caring versus controlling and holding evolved strategies. Frontiers10.3389/fpsyg.2020.582090 PMC7902494 33643109

[ref73] Gilbert P. Basran J. (2018). Imagining one’s compassionate self and coping with life difficulties. EC Psychol. Psychiatry 7, 971–978.

[ref74] Gilbert P. Basran J. (2019). The evolution of prosocial and antisocial competitive behavior and the emergence of prosocial and antisocial leadership styles. Front. Psychol. 10:610. 10.3389/fpsyg.2019.00610, PMID: 31293464 PMC6603082

[ref75] Gilbert P. Basran J. MacArthur M. Kirby J. N. (2019). Differences in the semantics of prosocial words: an exploration of compassion and kindness. Mindfulness 10, 2259–2271. 10.1007/s12671-019-01191-x

[ref76] Gilbert P. Broomhead C. Irons C. McEwan K. Bellew R. Mills A. (2007). Development of a striving to avoid inferiority scale. Br. J. Soc. Psychol. 46, 633–648. 10.1348/014466606X157789 17877856

[ref77] Gilbert P. Catarino F. Duarte C. Matos M. Kolts R. Stubbs J. (2017). The development of compassionate engagement and action scales for self and others. J. Compassionate Health Care 4, 1–24. 10.1186/s40639-017-0033-3

[ref78] Gilbert P. Choden (2013). Mindful compassion. London: Constable Robinson.

[ref79] Gilbert P. Irons C. (2005). “Focused therapies and compassionate mind training for shame and self-attacking” in Compassion: Conceptualisations, research and use in psychotherapy. ed. Gilbert P. (London: Routledge), 263–325.

[ref80] Gilbert P. Mascaro J. (2017). “Compassion: fears, blocks, and resistances: an evolutionary investigation” in The oxford handbook of compassion science. eds. Seppälä E. M. Simon-Thomas E. Brown S. L. Worline M. C. Cameron L. Doty J. R. (New York: Oxford University Press), 399–420.

[ref81] Gilbert P. McEwan K. Bellew R. Mills A. Gale C. (2009). The dark side of competition: how competitive behaviour and striving to avoid inferiority are linked to depression, anxiety, stress and self-harm. Psychol. Psychother. 82, 123–136. 10.1348/147608308X379806, PMID: 19040794

[ref82] Gilbert P. McEwan K. Catarino F. Baião R. Palmeira L. (2014). Fears of happiness and compassion in relationship with depression, alexithymia, and attachment security in a depressed sample. Br. J. Clin. Psychol. 53, 228–244. 10.1111/bjc.12037, PMID: 24283291

[ref83] Gilbert P. McEwan K. Matos M. Rivis A. (2011). Fears of compassion: development of three self-report measures. Psychol. Psychother. 84, 239–255. 10.1348/147608310X526511, PMID: 22903867

[ref84] Gilbert P. McEwan K. Mitra R. Franks L. Richter A. Rockliff H. (2008). Feeling safe and content: a specific affect regulation system? Relationship to depression, anxiety, stress, and self-criticism. J. Posit. Psychol. 3, 182–191. 10.1080/17439760801999461

[ref85] Gilbert P. Miles J. N. (2000). Sensitivity to social put-down: it’s relationship to perceptions of social rank, shame, social anxiety, depression, anger and self-other blame. Pers. Individ. Differ. 29, 757–774. 10.1016/s0191-8869(99)00230-5

[ref86] Gilbert P. Procter S. (2006). Compassionate mind training for people with high shame and self-criticism: a pilot study of a group therapy approach. Clin. Psychol. Psychother. 13, 353–379. 10.1002/cpp.507

[ref209] Gilbert P. Simos G. (in press). Compassion focused therapy: Clinical practice and applications. London: Routledge.

[ref87] Gillath O. Karantzas G. (2019). Attachment security priming: a systematic review. Curr. Opin. Psychol. 25, 86–95. 10.1016/j.copsyc.2018.03.001, PMID: 29621693

[ref88] Goetz J. E. Keltner D. Simon-Thomas E. (2010). Compassion: an evolutionary analysis and empirical review. Psychol. Bull. 136, 351–374. 10.1037/a0018807, PMID: 20438142 PMC2864937

[ref89] Goleman D. Davidson R. J. (2017). Altered traits: Science reveals how meditation changes your mind, brain, and body. New York: Penguin.

[ref210] Goodall J. (1990). Through a window: My thirty years with the chimpanzees of Gombe. London: Penguin.

[ref90] Gray J. A. (1987). The psychology of fear and stress. 2nd Edn. Cambridge: University Press.

[ref91] Green M. Kirby J. N. Nielsen M. (2018). The cost of helping: an exploration of compassionate responding in children. Br. J. Dev. Psychol. 36, 673–678. 10.1111/bjdp.12252, PMID: 29888498

[ref92] Greenberg L. (2003). Evolutionary perspectives on emotion: making sense of what we feel. J. Cogn. Psychother. 16, 331–348. 10.1891/jcop.16.3.331.52517

[ref93] Gu J. Strauss C. Bond R. Cavanagh K. (2015). How do mindfulness-based cognitive therapy and mindfulness-based stress reduction improve mental health and wellbeing? A systematic review and meta-analysis of mediation studies. Clin. Psychol. Rev. 37, 1–12. 10.1016/j.cpr.2015.01.006, PMID: 25689576

[ref94] Hanson R. (2020). Neurodharma: New science, ancient wisdom, and seven practices of the highest happiness. New York: Harmony.

[ref95] Harlow H. F. Mears C. (1979). The human model: Primate perspectives. Washington, DC: V.H. Winston.

[ref96] Hein G. Silani G. Preuschoff K. Batson C. D. Singer T. (2010). Neural responses to ingroup and outgroup members’ suffering predict individual differences in costly helping. Neuron 68, 149–160. 10.1016/j.neuron.2010.09.003, PMID: 20920798

[ref97] Heinrichs M. Baumgartner T. Kirschbaum C. Ehlert U. (2003). Social support and oxytocin interact to suppress cortisol and subjective response to psychosocial stress. Biol. Psychiatry 54, 1389–1398. 10.1016/s0006-3223(03)00465-7, PMID: 14675803

[ref98] Henrich J. (2020). The weirdest people in the world: How the west became psychologically peculiar and particularly prosperous. London: Penguin.

[ref99] Hermanto N. Zuroff D. C. (2016). The social mentality theory of self-compassion and self-reassurance: the interactive effect of care-seeking and caregiving. J. Soc. Psychol. 156, 523–535. 10.1080/00224545.2015.1135779, PMID: 26736073

[ref100] Hermanto N. Zuroff D. C. Kelly A. C. Leybman M. J. (2017). Receiving support, giving support, and self-reassurance: a daily diary test of social mentality theory. Pers. Individ. Differ. 107, 37–42. 10.1016/j.paid.2016.11.013

[ref101] Hofer M. A. (1984). Relationships as regulators: a psychobiologic perspective on bereavement. Psychosom. Med. 46, 183–197. 10.1097/00006842-198405000-00001, PMID: 6739679

[ref102] Hofer M. A. (1994). Early relationships as regulators of infant physiology and behavior. Acta Paediatr. 83, 9–18. 10.1111/j.1651-2227.1994.tb13260.x, PMID: 7981480

[ref103] Holmes J. (2001). The search for the secure base: Attachment theory and psychotherapy. UK: Routledge.

[ref104] Holmes J. Slade A. (2017). Attachment in therapeutic practice. London: Sage.

[ref105] Hornstein E. A. Eisenberger N. I. (2018). A social safety net: developing a model of social-support figures as prepared safety stimuli. Curr. Dir. Psychol. Sci. 27, 25–31. 10.1177/0963721417729036 27324266

[ref106] Hrdy S. B. (2009). Mothers and others: The evolutionary origins of mutual understanding. Cambridge, MA: Belknap/Harvard.

[ref107] Jazaieri H. Jinpa G. T. McGonigal K. Rosenberg E. Finkelstein J. Simon-Thomas E. (2013). Enhancing compassion: a randomized controlled trial of a compassion cultivation training program. J. Happiness Stud. 14, 1113–1126. 10.1007/s10902-012-9373-z

[ref108] Kelly A. C. Dupasquier J. (2016). Social safeness mediates the relationship between recalled parental warmth and the capacity for self-compassion and receiving compassion. Pers. Individ. Differ. 89, 157–161. 10.1016/j.paid.2015.10.017

[ref109] Kelly A. C. Zuroff D. C. Leybman M. J. Gilbert P. (2012). Social safeness, received social support, and maladjustment: testing a tripartite model of affect regulation. Cogn. Ther. Res. 36, 815–826. 10.1007/s10608-011-9432-5

[ref110] Keltner D. Kogan A. Piff P. K. Saturn S. R. (2014). The sociocultural appraisals, values, and emotions (SAVE) framework of prosociality: core processes from gene to meme. Annu. Rev. Psychol. 65, 425–460. 10.1146/annurev-psych-010213-115054 24405363

[ref111] Keltner D. Oatley K. Jenkins J. M. (2018). Understanding emotions. 4th Edn. London: Wiley Global Education.

[ref112] Kessler S. E. (2020). Why care: complex evolutionary history of human healthcare networks. Front. Psychol. 11:199. 10.3389/fpsyg.2020.00199, PMID: 32116974 PMC7031495

[ref113] Kim J. J. Kent K. M. Cunnington R. Gilbert P. Kirby J. N. (2020a). Attachment styles modulate neural markers of threat and imagery when engaging in self-criticism. Sci. Rep. 10:13776. 10.1038/s41598-020-70772-x, PMID: 32792601 PMC7426808

[ref114] Kim J. J. Parker S. Doty J. Cunnington R. Gilbert P. Kirby J. (2020b). Neurophysiological and behavioural markers of compassion. Sci. Rep. 10:6789. 10.1038/s41598-020-63846-3, PMID: 32322008 PMC7176659

[ref115] Kim J. J. Parker S. L. Henderson T. Kirby J. N. (2020c). Physiological fractals: visual and statistical evidence across timescales and experimental states. J. R. Soc. Interface 17:20200334. 10.1098/rsif.2020.0334, PMID: 32574539 PMC7328377

[ref116] Kirby J. N. Day J. Sagar V. (2019). The ‘Flow’of compassion: a meta-analysis of the fears of compassion scales and psychological functioning. Clin. Psychol. Rev. 70, 26–39. 10.1016/j.cpr.2019.03.001, PMID: 30884253

[ref117] Kirby J. Gilbert P. (2017). “The emergence of the compassion focused therapies” in Compassion: Concepts, research and applications. ed. Gilbert P. (London: Routledge), 258–285.

[ref118] Kumashiro M. Sedikides C. (2005). Taking on board liability-focused information: close positive relationship as a self-bolstering resource. Psychol. Sci. 16, 732–739. 10.1111/j.1467-9280.2005.01603.x, PMID: 16137260

[ref119] Kumsta R. (2019). The role of epigenetics for understanding mental health difficulties and its implications for psychotherapy research. Psychol. Psychother. 92, 190–207. 10.1111/papt.12227, PMID: 30924323

[ref231] Lama Dalai (1995). The power of compassion. India: Harper Collins.

[ref121] Lampert K. (2005). Traditions of compassion: From religious duty to social activism. New York: Palgrave Macmillan.

[ref122] Lawrence V. A. Lee D. (2014). An exploration of people’s experiences of compassion-focused therapy for trauma, using interpretative phenomenological analysis. Clin. Psychol. Psychother. 21, 495–507. 10.1002/cpp.1854, PMID: 23893917

[ref123] Lee D. (2005). “The perfect nurture a model to develop a compassionate mind within the context of cognitive therapy” in Compassion: Conceptualisations, research and use in psychotherapy. ed. Gilbert P. (London: Routledge), 326–351.

[ref124] Lewis C. S. (1961). A grief observed. London: Faber & Faber.

[ref125] Lippard E. T. Nemeroff C. B. (2020). The devastating clinical consequences of child abuse and neglect: increased disease vulnerability and poor treatment response in mood disorders. Am. J. Psychiatry 177, 20–36. 10.1176/appi.ajp.2019.19010020, PMID: 31537091 PMC6939135

[ref126] Lockwood P. L. Apps M. A. Chang S. W. (2020). Is there a ‘social’brain? Implementations and algorithms. Trends Cogn. Sci. 24, 802–813. 10.1016/j.tics.2020.06.011, PMID: 32736965 PMC7501252

[ref127] Loewenstein G. Small D. A. (2007). The scarecrow and the tin man: the vicissitudes of human sympathy and caring. Rev. Gen. Psychol. 11, 112–126. 10.1037/1089-2680.11.2.112

[ref232] Lunkenheimer E. Tiberio S. S. Skoranski A. M. Buss K. A. Cole P. M. (2018). Parent‐child coregulation of parasympathetic processes varies by social context and risk for psychopathology. Psychophysiology 55:e12985. 10.1111/psyp.12985, PMID: 28845519 PMC5773380

[ref128] Luyten P. Campbell C. Allison E. Fonagy P. (2020). The mentalizing approach to psychopathology: state of the art and future directions. Annu. Rev. Clin. Psychol. 16, 297–325. 10.1146/annurev-clinpsy-071919-015355, PMID: 32023093

[ref129] Margana L. Bhogal M. S. Bartlett J. E. Farrelly D. (2019). The roles of altruism, heroism, and physical attractiveness in female mate choice. Pers. Individ. Differ. 137, 126–130. 10.1016/j.paid.2018.08.018

[ref130] Marino L. (2017). Thinking chickens: a review of cognition, emotion, and behavior in the domestic chicken. Anim. Cogn. 20, 127–147. 10.1007/s10071-016-1064-4, PMID: 28044197 PMC5306232

[ref131] Marsh A. A. (2019). The caring continuum: evolved hormonal and proximal mechanisms explain prosocial and antisocial extremes. Annu. Rev. Psychol. 70, 347–371. 10.1146/annurev-psych-010418-103010, PMID: 30231001

[ref233] Mascaro J. S. Florian M. P. Ash M. J. Palmer P. K. Frazier T. Condon P.. (2020). Ways of knowing compassion: how do we come to know, understand, and measure compassion when we see it? Front. Psychol. 11:547241. 10.3389/fpsyg.2020.547241, PMID: 33132956 PMC7561712

[ref132] Mascaro J. Negi L. T. Raison C. (2017). “Cognitively based compassion training. Gleaning generalities from specific biological effects” in The Oxford handbook of compassion science. eds. Seppälä E. M. Simon-Thomas E. Brown S. L. Worline M. C. Cameron L. Doty J. R. (New York: Oxford University Press), 247–257.

[ref133] Matos M. Duarte J. Duarte C. Gilbert P. Pinto-Gouveia J. (2018). How one experiences and embodies compassionate mind training influences its effectiveness. Mindfulness 9, 1224–1235. 10.1007/s12671-017-0864-1

[ref134] Matos M. Duarte C. Duarte J. Pinto-Gouveia J. Petrocchi N. Basran J. (2017). Psychological and physiological effects of compassionate mind training: a pilot randomised controlled study. Mindfulness 8, 1699–1712. 10.1007/s12671-017-0745-7

[ref135] Mayseless O. (2016). The caring motivation: An integrated theory. Oxford: Oxford University Press.

[ref136] McGhee K. E. Bell A. M. (2014). Paternal care in a fish: epigenetics and fitness enhancing effects on offspring anxiety. Proc. Biol. Sci. 281:20141146. 10.1098/rspb.2014.1146, PMID: 25232132 PMC4211443

[ref137] McGilchrist I. (2018). Ways of attending: How our divided brain constructs the world. Oxon, UK: Routledge.

[ref138] Melis A. P. (2018). The evolutionary roots of prosociality: the case of instrumental helping. Curr. Opin. Psychol. 20, 82–86. 10.1016/j.copsyc.2017.08.019, PMID: 28850865

[ref139] Mikulincer M. Shaver P. R. (2016). Attachment in adulthood: Structure, dynamics, and change. 2nd Edn. New York: Guilford Press.

[ref234] Miller J. G. Kahle S. Troxel N. R. Hastings P. D. (2020). The development of generosity from 4 to 6 years: examining stability and the biopsychosocial contributions of children’s vagal flexibility and mothers’ compassion. Front. Psychol. 11:2988. 10.3389/fpsyg.2020.590384 PMC7674169 33224079

[ref235] Mobbs D. Marchant J. L. Hassabis D. Seymour B. Tan G. Gray M.. (2009). From threat to fear: the neural organization of defensive fear systems in humans. J. Neurosci. 29, 12236–12243. 10.1523/JNEUROSCI.2378-09.2009, PMID: 19793982 PMC2782300

[ref140] Mullen G. O’Reilly G. (2018). How can social mentality theory help us understand eating disorder presentations? A scoping review. J. Relat. Res. 9:e18. 10.1017/jrr.2018.17

[ref141] Muris P. Otgaar H. (2020). The process of science: a critical evaluation of more than 15 years of research on self-compassion with the self-compassion scale. Mindfulness 11, 1469–1482. 10.1007/s12671-020-01363-0

[ref142] Music G. (2017). Nurturing natures attachment and children’s emotional social cultural and brain development. 2nd Edn. Oxon, UK: Routledge.

[ref143] Music G. (2019). Nurturing children: From trauma to growth using attachment theory, psychoanalysis and neurobiology. London: Routledge.

[ref144] Narvaez D. (2017). “Evolution, child raising and compassionate morality” in *Compassion: Concepts,* research and applications. ed. Gilbert P. (London: Routledge), 31–68.

[ref211] Narvaez D. (2020). Ecocentrism: resetting baselines for virtue development. Ethical Theory Moral Pract. 23, 1–16. 10.1007/s10677-020-10091-2

[ref145] Neel R. Kenrick D. T. White A. E. Neuberg S. L. (2016). Individual differences in fundamental social motives. J. Pers. Soc. Psychol. 110, 887–907. 10.1037/pspp0000068, PMID: 26371400

[ref146] Neff K. D. (2020). Commentary on Muris and Otgaar (2020): let the empirical evidence speak on the self-compassion scale. Mindfulness 11, 1900–1909. 10.1007/s12671-020-01411-9

[ref147] Neff K. Germer C. (2017). “Self-compassion and psychological well-being” in The Oxford handbook of compassion science. eds. Seppälä E. M. Simon-Thomas E. Brown S. L. Worline M. C. Cameron L. Doty J. R. (New York: Oxford University Press), 371–386.

[ref148] Nesse R. M. (2019). Good reasons for bad feelings: Insights from the frontier of evolutionary psychiatry. New York: Dutton.

[ref237] Nguyen T. Schleihauf H. Kayhan E. Matthes D. Vrtička P. Hoehl S. (2020). The effects of interaction quality on neural synchrony during mother-child problem solving. Cortex 124, 235–249. 10.1016/j.cortex.2019.11.020, PMID: 31927470

[ref149] Norman L. Lawrence N. Iles A. Benattayallah A. Karl A. (2015). Attachment-security priming attenuates amygdala activation to social and linguistic threat. Soc. Cogn. Affect. Neurosci. 10, 832–839. 10.1093/scan/nsu127, PMID: 25326039 PMC4448028

[ref150] Overmier J. B. (2002). On learned helplessness. Integr. Physiol. Behav. Sci. 37, 4–8. 10.1007/BF02688801, PMID: 12069364

[ref151] Pace T. W. Negi L. T. Adame D. D. Cole S. P. Sivilli T. I. Brown T. D.. (2009). Effect of compassion meditation on neuroendocrine, innate immune and behavioral responses to psychosocial stress. Psychoneuroendocrinology 34, 87–98. 10.1016/j.psyneuen.2008.08.011, PMID: 18835662 PMC2695992

[ref152] Panksepp J. (1998). Affective neuroscience. New York: Oxford University Press.

[ref153] Pauley G. McPherson S. (2010). The experience and meaning of compassion and self-compassion for individuals with depression or anxiety. Psychol. Psychother. 83, 129–143. 10.1348/147608309X471000, PMID: 19785933

[ref154] Perry R. Sibley G. Duckitt J. (2013). Dangerous and competitive worldviews: a meta-analysis of their associations with social dominance orientation and right-wing authoritarianism. J. Res. Pers. 47, 116–127. 10.1016/j.jrp.2012.10.004

[ref155] Petrocchi N. Cheli S. (2019). The social brain and heart rate variability: implications for psychotherapy. Psychol. Psychother. 92, 208–223. 10.1111/papt.12224 30891894

[ref156] Piff P. K. Kraus M. W. Keltner D. (2018). Unpacking the inequality paradox: the psychological roots of inequality and social class. Adv. Exp. Soc. Psychol. 57, 53–124. 10.1016/bs.aesp.2017.10.002

[ref157] Porges S. W. (2007). The polyvagal perspective. Biol. Psychol. 74, 116–143. 10.1016/j.biopsycho.2006.06.009, PMID: 17049418 PMC1868418

[ref158] Porges S. W. (2017). “Vagal pathways: portals to compassion” in The Oxford handbook of compassion science. eds. Seppälä E. M. Simon-Thomas E. Brown S. L. Worline M. C. (New York: Oxford University Press).

[ref159] Porges S. W. Furman S. A. (2011). The early development of the autonomic nervous system provides a neural platform for social behaviour: a polyvagal perspective. Infant Child Dev. 20, 106–118. 10.1002/icd.688, PMID: 21516219 PMC3079208

[ref160] Poulin M. J. (2017). “To help or not to help: goal commitment and the goodness of compassion” in The Oxford handbook of compassion science. eds. Seppälä E. M. Simon-Thomas E. Brown S. L. Worline M. C. Cameron L. Doty J. R. (New York: Oxford University Press), 355–367.

[ref161] Preston S. D. (2013). The origins of altruism in offspring care. Psychol. Bull. 139, 1305–1341. 10.1037/a0031755, PMID: 23458432

[ref162] Rachman S. J. (1990). Fear and courage. New York: WH Freeman/Times Books/Henry Holt & Co.

[ref163] Ricard M. (2015). Altruism: The power of compassion to change itself and the world. Great Britain: Atlantic Books.

[ref164] Richins M. T. Barreto M. Karl A. Lawrence N. (2019). Empathic responses are reduced to competitive but not non-competitive outgroups. Soc. Neurosci. 14, 345–358. 10.1080/17470919.2018.1463927, PMID: 29633906

[ref165] Riskind J. H. Rector N. A. Taylor S. (2012). Looming cognitive vulnerability to anxiety and its reduction in psychotherapy. J. Psychother. Integr. 22, 137–162. 10.1037/a0028011

[ref167] Rothschild B. (2000). The body remembers: The psychophysiology of trauma & trauma treatment. New York: WW Norton & Company.

[ref168] Ryan C. (2019). Civilized to death: The price of progress. New York: Avid Reader Press/Simon & Schuster.

[ref169] Sachs J. D. (2012). The price of civilization: Reawakening American virtue and prosperity. London: Random House.

[ref236] Sapolsky M. R. (2017). Behave: The biology of humans at our best and worst. London: Vintage.

[ref170] Sapolsky R. M. Share L. J. (2004). A pacific culture among wild baboons: its emergence and transmission. PLoS Biol. 2:E106. 10.1371/journal.pbio.0020106, PMID: 15094808 PMC387274

[ref171] Schore A. N. (2019). Right brain psychotherapy. New York: Norton.

[ref172] Seppälä E. M. Simon-Thomas E. Brown S. L. Worline M. C. Cameron C. D. Doty J. R. (eds.) (2017). The Oxford handbook of compassion science. New York: Oxford University Press.

[ref173] Shirtcliff E. A. Vitacco M. J. Graf A. R. Gostisha A. J. Merz J. L. Zahn-Waxler C. (2009). Neurobiology of empathy and callousness: implications for the development of antisocial behavior. Behav. Sci. Law 27, 137–171. 10.1002/bsl.862, PMID: 19319834 PMC2729461

[ref174] Siegel D. J. (2010). The mindful therapist: A clinician’s guide to mindsight and neural integration. New York: WW Norton.

[ref175] Siegel D. J. (2012). The developing mind: How relationships and the brain interact to shape who we are. New York: Guilford Press.

[ref176] Siegel D. J. (2019). The mind in psychotherapy: an interpersonal neurobiology framework for understanding and cultivating mental health. Psychol. Psychother. 92, 224–237. 10.1111/papt.12228, PMID: 31001926

[ref177] Singer T. Engert V. (2019). It matters what you practice: differential training effects on subjective experience, behavior, brain and body in the ReSource project. Curr. Opin. Psychol. 28, 151–158. 10.1016/j.copsyc.2018.12.005, PMID: 30684917

[ref178] Slavich G. M. (2020). Social safety theory: a biologically based evolutionary perspective on life stress, health, and behavior. Annu. Rev. Clin. Psychol. 16, 265–295. 10.1146/annurev-clinpsy-032816-045159, PMID: 32141764 PMC7213777

[ref179] Spikins P. (2015). How compassion made us human: The evolutionary origins of tenderness trust and morality. Barnsley: Spear and Sword Books.

[ref238] Spikins P. (2017). “Prehistoric origins the compassion of far distant strangers” in Compassion: Concepts, research and applications. ed. Gilbert P. (London: Routledge), 16–30.

[ref180] Steffen P. R. Foxx J. Cattani K. Alldredge C. Austin T. Burlingame G. M. (2020). Impact of a 12-week group-based compassion focused therapy intervention on heart rate variability. Appl. Psychophysiol. Biofeedback. 10.1007/s10484-020-09487-8, PMID: [Epub ahead of print] 32939617

[ref181] Stellar J. E. Keltner D. (2017). “Compassion in the autonomic nervous system: the role of the vagus nerve” in Compassion: Concepts, research and applications. ed. Gilbert P. (London: Routledge), 120–134.

[ref182] Stevens J. Woodruff C. C. (2018). The neuroscience of empathy, compassion and self-compassion. London: Academic Press.

[ref183] Strauss C. Taylor B. L. Gu J. Kuyken W. Baer R. Jones F.. (2016). What is compassion and how can we measure it? A review of definitions and measures. Clin. Psychol. Rev. 47, 15–27. 10.1016/j.cpr.2016.05.004, PMID: 27267346

[ref184] Suddendorf T. (2018). “Two key features created the human mind: inside our heads” in Scientific American. Vol. 319 42–47.29924104

[ref185] Suddendorf T. Whitten A. (2001). Mental evolutions and development: evidence for secondary representation in children, great apes, and other animals. Psychol. Bull. 127, 629–650. 10.1037/0033-2909.127.5.629, PMID: 11548971

[ref186] Taylor C. (1989). Sources of the self: The making of the modern identity. Cambridge: Harvard University Press.

[ref187] Thayer J. F. Åhs F. Fredrikson M. Sollers J. J. III Wager T. D. (2012). A meta-analysis of heart rate variability and neuroimaging studies: implications for heart rate variability as a marker of stress and health. Neurosci. Biobehav. Rev. 36, 747–756. 10.1016/j.neubiorev.2011.11.009, PMID: 22178086

[ref188] Tost H. Kolachana B. Hakimi S. Lemaitre H. Verchinski B. A. Mattay V. S.. (2010). A common allele in the oxytocin receptor gene (OXTR) impacts prosocial temperament and human hypothalamic-limbic structure and function. Proc. Natl. Acad. Sci. U. S. A. 107, 13936–13941. 10.1073/pnas.1003296107, PMID: 20647384 PMC2922278

[ref189] Twenge J. M. Miller J. D. Campbell W. K. (2014). The narcissism epidemic: commentary on modernity and narcissistic personality disorder. Pers. Disord. 5, 227–229. 10.1037/per0000008, PMID: 24796568

[ref190] Uomini N. Fairlie J. Gray R. D. Griesser M. (2020). Extended parenting and the evolution of cognition. Philos. Trans. R. Soc. Lond. B Biol. Sci. 375:20190495. 10.1098/rstb.2019.0495, PMID: 32475334 PMC7293161

[ref191] Van der Kolk B. A. (2015). The body keeps the score: Brain, mind, and body in the healing of trauma. UK: Penguin Books.

[ref192] Wang S. (2005). “A conceptual framework for integrating research relatedto the physiology of compassion and the wisdom of Buddhist teachings” in Compassion: Conceptualizations, research and use in psychotherapy. ed. Gilbert P. (London: Routledge), 75–120.

[ref193] Weiss E. Zaki J. (2017). “Empathy building interventions: a review of existing work and suggestions for future directions” in The Oxford handbook of compassion science. eds. Seppälä E. M. Simon-Thomas E. Brown S. L. Worline M. C. Cameron L. Doty J. R. (New York: Oxford University Press), 399–420.

[ref194] Weng H. Y. Fox A. S. Shackman A. J. Stodola D. E. Caldwell J. Z. Olson M. C.. (2013). Compassion training alters altruism and neural responses to suffering. Psychol. Sci. 24, 1171–1180. 10.1177/0956797612469537, PMID: 23696200 PMC3713090

[ref195] Weng H. Y. Lapate R. C. Stodola D. E. Rogers G. M. Davidson R. J. (2018). Visual attention to suffering after compassion training is associated with decreased amygdala responses. Front. Psychol. 9:771. 10.3389/fpsyg.2018.00771, PMID: 29872413 PMC5972817

[ref196] Wetherall K. Robb K. A. O’Connor R. C. (2019). Social rank theory of depression: a systematic review of self-perceptions of social rank and their relationship with depressive symptoms and suicide risk. J. Affect. Disord. 246, 30–39. 10.1016/j.jad.2018.12.045, PMID: 30594043

[ref197] Workman L. Reader W. Barkow J. H. (2020). The Cambridge handbook of evolutionary perspectives on human behavior. Cambridge: Cambridge University Press.

[ref198] Wright N. Hill J. Sharp H. Pickles A. (2018). Maternal sensitivity to distress, attachment and the development of callous-unemotional traits in young children. J. Child Psychol. Psychiatry 59, 790–800. 10.1111/jcpp.12867, PMID: 29380375 PMC6033174

[ref199] Zuroff D. C. Clegg K. A. Levine S. L. Hermanto N. Armstrong B. F. Haward B. (in press). Beyond trait models of self-criticism and self-compassion: variability over domains and the search for signatures.

